# Genome-wide identification and characterization of toll-like receptor 5 (*TLR5*) in fishes

**DOI:** 10.3389/fgene.2022.1083578

**Published:** 2023-01-06

**Authors:** Kai Zhang, Ming Chen, Haobin He, Hongyan Kou, Li Lin, Rishen Liang

**Affiliations:** ^1^ College of Animal Science and Technology, Zhongkai University of Agriculture and Engineering, Guangzhou, China; ^2^ Guangdong Provincial Water Environment and Aquatic Products Security Engineering Technology Research Center, Guangzhou, China

**Keywords:** TLR5, fish, whole-genome duplications, gene loss, adaptive evolution

## Abstract

Toll-like receptors 5 (TLR5), a member of the toll-like receptors (TLRs) family, is a class of pattern recognition receptors (PRRs) that recognize pathogen-associated molecular patterns (PAMPs). It responds to vertebrate recognition of bacterial flagellin and participates in innate immune responses. However, genome-wide identification and characterization of TLR5 in fishes have not been investigated. Here, three TLR5M isotypes (*TLR5Ma*, *TLR5Mb1*, and *TLR5Mb2*) and a TLR5S are all extracted from fish genomes on the basis of phylogenetic and synteny analyses. We confirmed that the non-teleost fishes have one *TLR5M* gene, as well as additional *TLR5* genes (*TLR5M* and *TLR5S*) in teleost fishes. In addition, some special teleost fishes possess two to three *TLR5* genes, which have undergone the fourth whole-genome duplication (WGD). According to our results, we inferred that the diversity of *TLR5* genes in fishes seems to be the result of combinations of WGD and gene loss. Furthermore, *TLR5* isoforms displayed differences at the flagellin interaction sites and viral binding sites, and showed lineage-specific, which indicated that *TLR5* duplicates may generate functional divergence. Bacterial experiments also supported the idea that *CiTLR5Ma* and *CiTLR5Mb* are subfunctionalized to sense bacterial flagellin. In summary, our present comparative genomic survey will benefit for further functional investigations of *TLR5* genes in fish.

## 1 Introduction

In the innate immune landscape, toll-like receptors (TLRs) play a major role by activating the first line of defense against invading microbial pathogens in invertebrate and vertebrate lineages (Purcell et al., 2006; Brikos and O'Neill, 2008). TLRs are antigen-recognition receptors on the surfaces or inside cells that directly recognize pathogen-associated molecular patterns (PAMPs) (Mogensen, 2009). All TLRs have a leucine-rich repeat (LRR) ectodomain for PAMP recognition, transmembrane (TM) domain for TLR dimerization and stabilization, and toll/IL-1 receptor (TIR) domain associated with signaling (Brubaker et al., 2015; Voogdt et al., 2016; Voogdt et al., 2018; Su and Yu, 2019; Gao et al., 2022; Liao et al., 2022). According to the previous data, at least 13 TLR types have been reported in mammals (Janeway and Medzhitov, 2002; Akira et al., 2006); for example, 12 (*TLR1*–*9*, *TLR11*–*13*) and 10 (*TLR1*–*10*) *TLR* genes have been reported in the mice and human genome, respectively. Moreover, 22 *TLR* genes were identified in the fish genome (Palti, 2011). Furthermore, fish-specific TLRs (soluble *TLR5*, *TLR14*, *TLR18*–*20*, and *TLR22*–*28*) and mammalian TLR orthologs (*TLR1*, *TLR2*, *TLR3*, *TLR5*, *TLR7*, *TLR8*, and *TLR9*) are included in the teleost fish TLRs (Matsuo et al., 2008; Palti, 2011).

As a TLR family member, TLR5 is a critical factor in initiating the innate immune response and triggering adaptive immunity (Hayashi et al., 2001; Didierlaurent et al., 2004), which can activate flagellin-mediated NF-κB via the MyD88-dependent pathway in the cellular membrane (Mizel et al., 2003). Previously, TLR5 in teleosts was divided into the membrane and soluble forms of TLR5 (*TLR5M* and *TLR5S*), respectively (Oshiumi et al., 2003; Tsujita et al., 2004), which are reported in several fishes, including orange-spotted grouper (*Epinephelus coioides*) (Bai et al., 2017), rainbow trout (*Oncorhynchus mykiss*) (Tsujita et al., 2004), fugu (*Fugu rubripes*) (Oshiumi et al., 2003), Japanese flounder (*Paralichthys olivaceus*) (Hwang et al., 2010), and golden pompano (*Trachinotus ovatus*) (Zhu et al., 2020). Recent studies reported that *TLR5M* and *TLR5S* play vital roles in TLR/IL-1R signaling pathways and immune response to the invasions of a broad range of pathogens in fish. Furthermore, *TLR5M* stimulation with *Vibrio anguillarum* or its flagellin possibly activated *TLR5S* expression. Moreover, TLR5M signaling was amplified in rainbow trout flagellin through interaction with TLR5S in positive loop feedback (Tsujita et al., 2004). In addition, studies revealed different TLR5M types in turbot and blunt snout bream, namely *TLR5a* and *TLR5b*, respectively (Liu et al., 2017; Zhan et al., 2019).

Although there were reports of TLR5 types in several bony fishes (Jiang et al., 2015; Han et al., 2017; Gao et al., 2022), the molecular evolution and a comprehensive comparative genomic survey of these gene families in different fishes have not yet been reported. Rapid advancement in sequencing technologies has enabled access to high-quality whole-genome data of many fish species, thus allowing for the extraction of fish *TLR5* genes and their encoding sequences, including, tetraploid *Sinocyclocheilus* fishes, amphibious mudskippers, salmonids, deep-sea snailfish, cartilaginous sharks, lobe-finned fish (coelacanth), hagfish, and jawless sea lamprey. This enables the study of the presence or absence of species-specific *TLR5* isotypes and differences (variations) in sequences across different species. This study investigated fish *TLR5* genes and protein structures after extracting nucleotide sequences. Afterward, phylogenetic and synteny analyses were performed. Moreover, the expression patterns and primary functions of the fish *TLR5* genes after bacterial infection were identified. These results are valuable for future research and lay a solid foundation for investigating the mechanisms underlying fish *TLR5* genes.

## 2 Materials and methods

### 2.1 Acquisition of TLR5 for nucleotide and protein sequences

A total of 30 species of fish were included in this research. Two methods were used to obtain data; first, the unreported *TLR5* sequences from 28 species of fish were used and obtained from our complete genomic data (Table 1). Second, Ensembl (https://asia.ensembl.org/index.html) and GenBank (https://www.ncbi.nlm.nih.gov/genbank/) public databases were utilized for the purpose of downloading published *TLR5* sequence data. Through tBLASTn (https://blast.ncbi.nlm.nih.gov/Blast.cgi), the prospective homology-based *TLR5* genes were recovered from fish genomes (Pevsner, 2009), with an e-value of 10^–5^. The best hit for each alignment was found using the BLAST results treated with the Perl script. Finally, the *TLR5* genes were predicted from the best hits using GeneWise v2.2.0 (Birney et al., 2004).

**TABLE 1:** Copy numbers of TLR5 genes in the examined fish genomes.

Class	Common name	Species name (with an abbreviation)	Ploidy	Total	TLR5S	TLR5Ma	TLR5Mb
Actinopterygii	Large yellow croaker	*Larimichthys crocea* (lac)	diploid	2	1	1	0
	Japanese medaka	*Oryzias latipes* (orl)	diploid	2	1	1	0
	Indian medaka	*Oryzias melastigma* (inm)	diploid	2	1	1	0
	Tilapia	*Oreochromis mossambicus* (til)	diploid	2	1	1	0
	Zig Zag Eel	*Mastacembelus armatus* (zze)	diploid	2	1	1	0
	black porgy	*Acanthopagrus schlegelii* (blp)	diploid	1	0	1	0
	torafugu	*Takifugu rubripes* (fugu)	diploid	1	0	1	0
	Japanese flounder	*Paralichthys olivaceus* (pao)	diploid	2	1	1	0
	three-spined stickleback	*Gasterosteus aculeatus* (sti)	diploid	1	0	1	0
	tiger tail seahorse	*Hippocampus comes* (tts)	diploid	2	1	1	0
	Tanaka’s snailfish	*Liparis tanakae* (lit)	diploid	1	0	1	0
	Mariana hadal snailfish	*Pseudoliparis swirei* (pss)	diploid	1	0	1	0
	spotted gar	*Lepisosteus oculatus* (spg)	diploid	1	0	1	0
	Atlantic salmon	*Salmo salar* (sas)	tetraploid	3	1	1	1
	river trout	*Salmo trutta* (sat)	tetraploid	3	1	1	1
	sockeye salmon	*Oncorhynchus nerka* (sos)	tetraploid	3	1	1	1
	rainbow trout	*Oncorhynchus mykiss* (rat)	tetraploid	3	1	1	1
	Chinook salmon	*Oncorhynchus tshawytscha* (chs)	tetraploid	3	1	1	1
	pink salmon	*Oncorhynchus gorbuscha* (pis)	tetraploid	3	1	1	1
	chum salmon	*Oncorhynchus keta* (csa)	tetraploid	3	1	1	1
	common carp	*Cyprinus carpio* (coc)	tetraploid	2	0	1	1
	grass carp	*Ctenopharyngodon idella* (grc)	tetraploid	2	0	1	1
	blunt snout bream	*Megalobrama amblycephala* (bsb)	tetraploid	2	0	1	1
	Goldfish	*Carassius auratus* (gof)	tetraploid	2	0	1	1
	Golden-line barbel fishes	*Sinocyclocheilus grahami* (sgr)	tetraploid	3	0	1	2 (5Mb1,b2)
		*Sinocyclocheilus rhinocerous* (srh)	tetraploid	3	0	1	2 (5Mb1,b2)
		*Sinocyclocheilus anshuiensis* (san)	tetraploid	3	0	1	2 (5Mb1,b2)
		*Sinocyclocheilus maitianheensis* (sma)	tetraploid	3	0	1	2 (5Mb1,b2)
Sarcopterygii	Coelacanth	*Latimeria chalumnae* (coe)	diploid	1	0	1	0
Chondrichthyes	elephant shark	*Callorhinchus milii* (cam)	diploid	1	0	1	0
Agnatha	sea lamprey	*Petromyzon marinus* (pem)	diploid	1	0	1	0

### 2.2 Phylogenetic analysis and sequence alignment

Phylogenetic analysis was conducted by utilizing nucleotide and protein sequences of all *TLR5* genes. Protein sequences of TLR5 were aligned using the MAFFT software (Yamada et al., 2016), and RAxML8.0.17 was employed to conduct a maximum likelihood (ML) phylogenetic analysis (Stamatakis, 2006; Stamatakis et al., 2008). The FastTree v2.1.7 software was employed to generate the ML phylogenetic trees of the *TLR5* isotypes based on their corresponding coding sequences (Price et al., 2009). In addition, a protein model of human TLR5M was downloaded from the public Protein DataBank to compare the structural differences of fish TLR5 proteins.

### 2.3 Analyses of conserved synteny

The conservation of *TLR5* genes was assessed by observing the genes in the up and downstream regions of each *TLR5M* and *TLR5S* paralog. Moreover, associated genomic data was also obtained from GenBank and our lab. The genome of zebrafish was considered the reference standard for any down and upstream regions of *TLR5*.

### 2.4 Experimental fish

Healthy and juvenile *Ctenopharyngodon idellus* (80 ± 20 g) were purchased from a farm in Guangdong Province, China. The fish were kept at 25°C–26°C for 2 weeks in a flow-through water system to ensure their acclimatization to the laboratory conditions before the experiments.

### 2.5 Bacterail challenge and sample collection

Luria-Bertani (LB) broth was used for the culturing of *Aeromonas hydrophila* (ZK2022061) at a temperature of 37°C and utilized for immune system challenges. After 12 h, bacteria were washed twice with sterile phosphate-buffered saline (PBS), and the bacterial concentration was adjusted to 3 × 10^7^ colony-forming units/mL. *C. idellus* was divided into *A. hydrophila* and PBS control groups. Each fish was injected with 100 μl of the bacterial suspension or PBS (control group). At time intervals of 0, 6, 9, 12, 24, and 48 h after infection, three replicates of the fish’s important immune organs (head kidney, liver, and spleen) were collected from the groups, immediately frozen in liquid nitrogen, and stored at −80°C.

### 2.6 RNA extraction, cDNA synthesis and qPCR analysis

The total RNA was extracted using TRIzol (Life Technologies, California, United States), synthesized to the first-strand cDNA using HIScript Q Select RT SuperMix (Vazyme, Nanjing, China), and stored at -20°C for qRT-PCR detection. All steps were performed according to the manufacturer’s instructions. The five-fold dilution of cDNA templates was carried out, and β-actin was employed as the internal control. Using the obtained sequences of *C. idellus*, the primers of the gene of interest were designed using Primer Premier 6.0, and the primers of β-actin were used herein. All primers are presented in Supplementary Table S1. The total reaction volume of 20 μl was designed as follows: 4 μl diluted cDNA, 0.5 μl of each specific primer, 10 μl AceQ qPCR SYBR Green Master Mix (Vazyme, Nanjing, China), and 5 μl Diethyl Pyrocarbonate (DEPC) water. The amplification was performed under the following conditions: 5 min at 95°C, 10 s at 95°C (40 cycles), and 30 s at 60°C (40 cycles). The experiment was conducted in triplicates, and the relative levels of expression of the target genes were calculated by employing the 2^−ΔΔCT^ method (Schmittgen and Livak, 2008). The discrepancy between different treatments was analyzed using the one-way analysis of variance (ANOVA), and *p* < 0.05 or *p* < 0.01 was considered statistically significant.

## 3 Results

### 3.1 Copy number variation

In total, 40 vertebrate species (Table 1) were studied to collect the TLR5 sequences, and only a single *TLR5* gene was identified among the genomes of Chondrichthyes, Sarcopterygii, reptiles, amphibians, and mammals. According to previous reports, teleosts contain two *TLR5* genes (Oshiumi et al., 2003); however, the present study confirmed that the teleosts contain four *TLR5* genes. One or two *TLR5* genes were present in the common diploid teleost, which is consistent with previous findings (Oshiumi et al., 2003; Zhu et al., 2020). The economically important Tilapia (*Oreochromis mossambicus*) and large yellow croaker (*Larimichthys crocea*) possessed two TLR5 forms, TLR5M and TLR5S (Table 1). Interestingly, in Black porgy (*Acanthopagrus schlegelii*) and Mariana hadal snailfish (*Pseudoliparis swirei*), only *TLR5M* was observed while *TLR5S* was absent. Two copies of the *TLR5M* gene, temporally named *TLR5Ma* and *TLR5Mb*, and one isotype, *TLR5S*, were observed in the typical tetraploid teleost, such as Atlantic salmon (*Salmo salar*) and rainbow trout (*Oncorhynchus mykiss*), which experienced the salmonid-specific genome duplication. In tetraploid Cyprinidae, which underwent carp-specific genome duplication, two copies of *TLR5* genes, *TLR5Ma* and *TLR5Mb*, were observed, with no *TLR5S* gene. However, the tetraploid *Sinocyclocheilus* fish possessed double copies of the *TLR5Mb* gene, temporally named *TLR5Mb1* and *TLR5Mb2*, and one *TLR5Ma* gene.

### 3.2 Phylogenetic relationships

**FIGURE 1:**
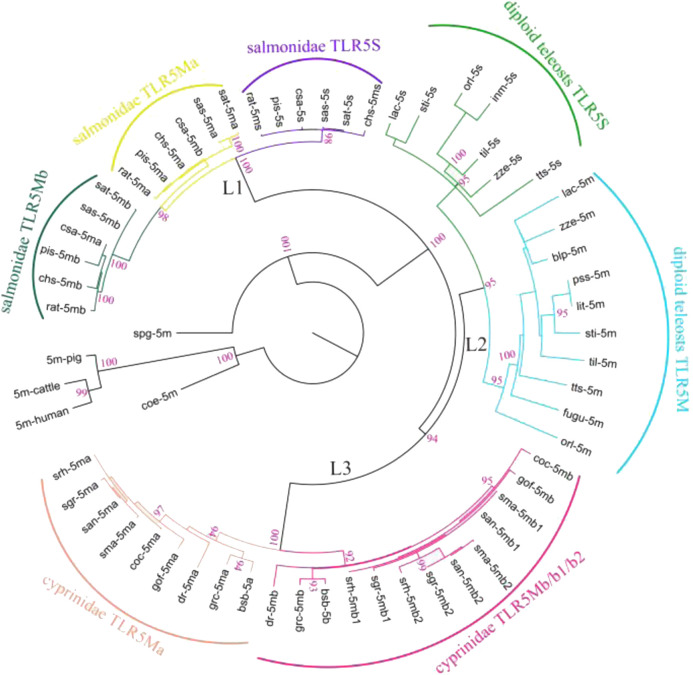
Phylogenetic relationships among various fish *TLR5s*. Representative *TLR5* genes were categorized into three main clades (L1, L2, and L3). Furthermore, diploid teleost *TLR5* genes were classified into two subgroups (diploid teleost TLR5S and TLR5M). *TLR5* genes in Salmonidae were clustered into three different clades (salmonidae TLR5S, TLR5Mb, and TLR5Mb). *TLR5* genes in Cyprinidae were divided into two subgroups (cyprinidae TLR5Ma and TLR5Mb/b1/b2). Detailed abbreviation of each species is available in Table 1.

A phylogenetic analysis was performed using protein sequences of all *TLR5* genes (Figure 1). These TLR5 proteins were divided into three main clades with high support values (L1, L2, and L3). In the ancient spotted garfish (*Lepisosteus oculatus*), TLR5M was located out of the whole teleost TLR5 clade, whereas a single *TLR5* gene in ancient coelacanth fish (*Latimeria chalumnae*) was located within the mammalian clade. Furthermore, diploid teleost *TLR5* genes were classified into *TLR5M* and *TLR5S* subgroups, consistent with the teleost experiencing the third teleost-specific WGD. *TLR5Ma*, *TLR5Mb*, and *TLR5S* in Protacanthopterygii, including rainbow trout, and Atlantic salmon diverged into three different clades. In Cyprinidae, such as zebrafish, grass carp, common carp, and *Sinocyclocheilus*, the *TLR5* genes comprised the L3 clade, which was also divided into TLR5Ma and TLR5Mb/b1/b2 subgroups.

### 3.3 Synteny data

**FIGURE 2:**
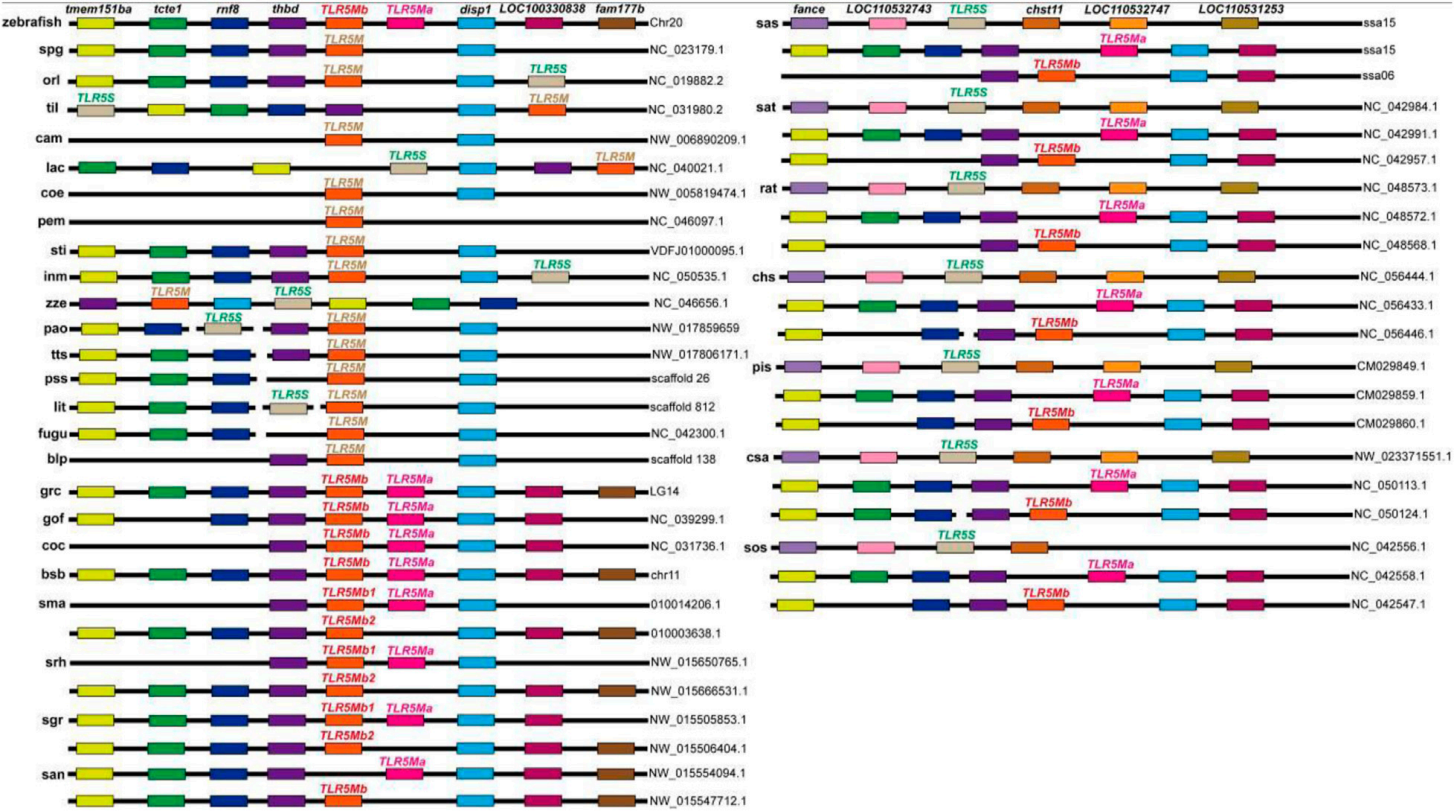
Synteny analyses of the chromosome loci harboring *TLR5* genes in fishes. Different colored rectangles represent different gene loci. Some gene loci near *TLR5* present conserved synteny in these examined fishes. Detailed abbreviation of each species is available in Table 1.

For the assessment of the chromosomal or scaffold synteny, *in silico* searches were carried out for conserved genes downstream and upstream of the *TLR5* isotypes in various fishes. It was observed that all *TLR5* genes shared a conserved suite of genes binding them on their side (Figure 2); however, several species displayed gene loss. Interestingly, *TLR5Ma* and *TLR5Mb/b1* of Cyprinidae, such as zebrafish, grass carp, common carp, and *Sinocyclocheilus*, were closely located within the same chromosome, whereas *TLR5Mb2* of *Sinocyclocheilus* was located on another chromosome. Moreover, *TLR5S* and *TLR5M* reside on the same chromosome or scaffold with minor variations in the location in some diploid teleost, including medaka and tilapia. Furthermore, the collinear analysis also established the reliability of the extracted *TLR5* genes.

### 3.4 The structure of TLR5 proteins

**FIGURE 3:**
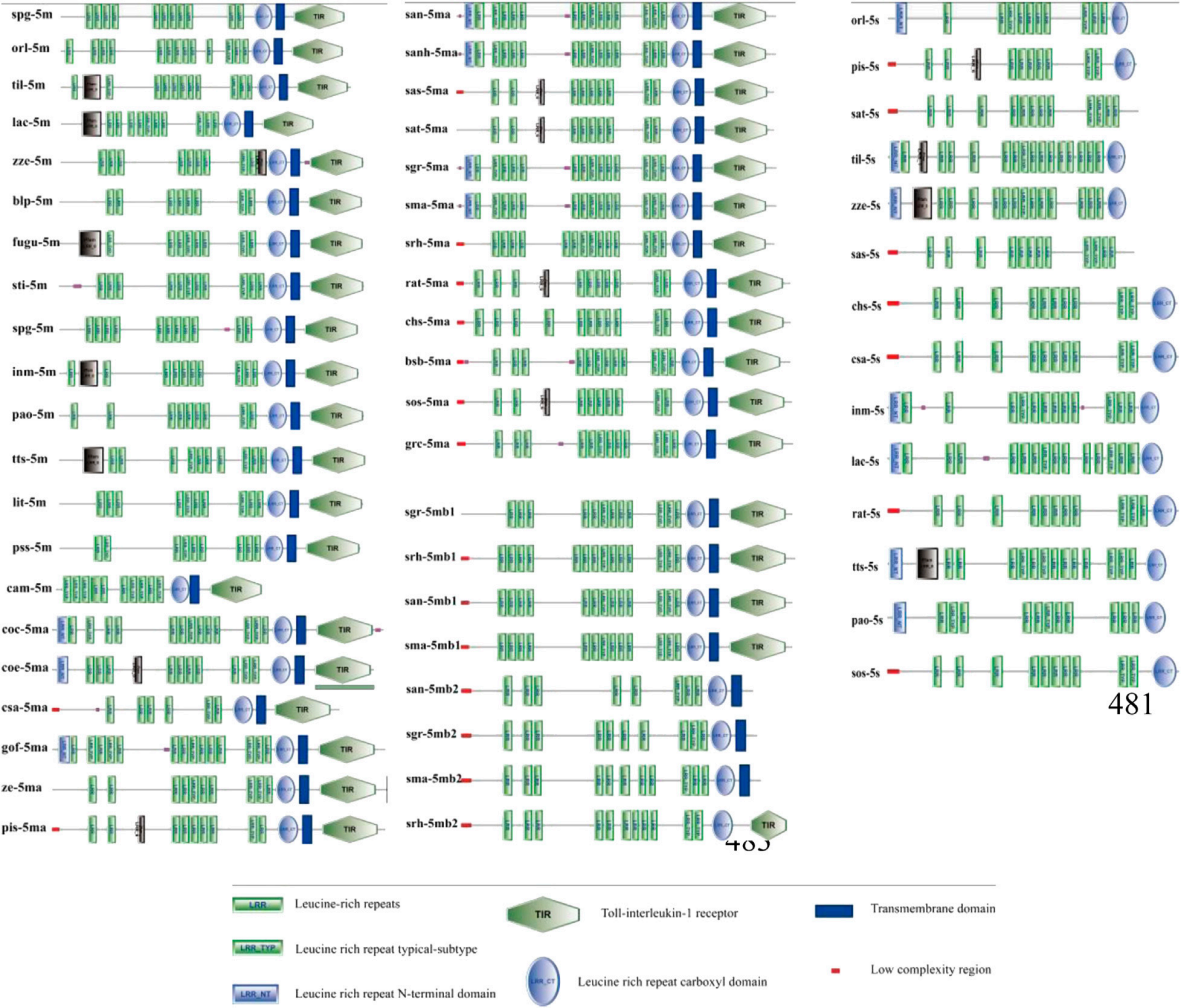
The domain features of *TLR5* genes among fishes. Detailed abbreviation of each species is available in Table 1.

The domain features of the TLR5 types were analyzed among various fishes (Figure 3). The domains of the representative fish TLR5M members, such as TLR5Ma and TLR5Mb/b1, contained three domains, including the LRR ectodomain, TM domain, and TIR domain. However, *Sinocyclocheilus* TLR5Mb2 consisted of only the LRR and TM domains. Fish TLR5S only contained the LRR domains. Moreover, the number of LRR domains varied not only across different TLR5 types of a single species but also across different fish species.

Representative TLR5 protein sequences were aligned to check the secondary structures of these TLR5 types in fishes (Supplementary Figure S1). The research on amino acid sites associated with the functional and structural features of TLR5 proteins was carried out. At the two disulfide bonds (583–610 and 585–629 residues), most residues were well conserved among these TLR5 sequences. Some residues around the glycosylation sites, including 37, 46, 245, 342, 422, 595, and 598, were non-conserved. Furthermore, three residues (294, 366, and 342) were responsible for the flagellin interaction; however, one of them showed differences among the fish TLR5 isoforms, such as D294-S294-N294. In the TIR domain, two significant residues, including Pro736 and Tyr798, were well conserved. Pro736 binds to the signaling adaptor molecule MyD88, and Tyr798 phosphorylates upon flagellin recognition. In addition, the amino acid difference was identified in the TLR5 residue 268 (G268S).

### 3.5 Tissue expression of *CiTLR5Ma* and *CiTLR5Mb*

**FIGURE 4:**
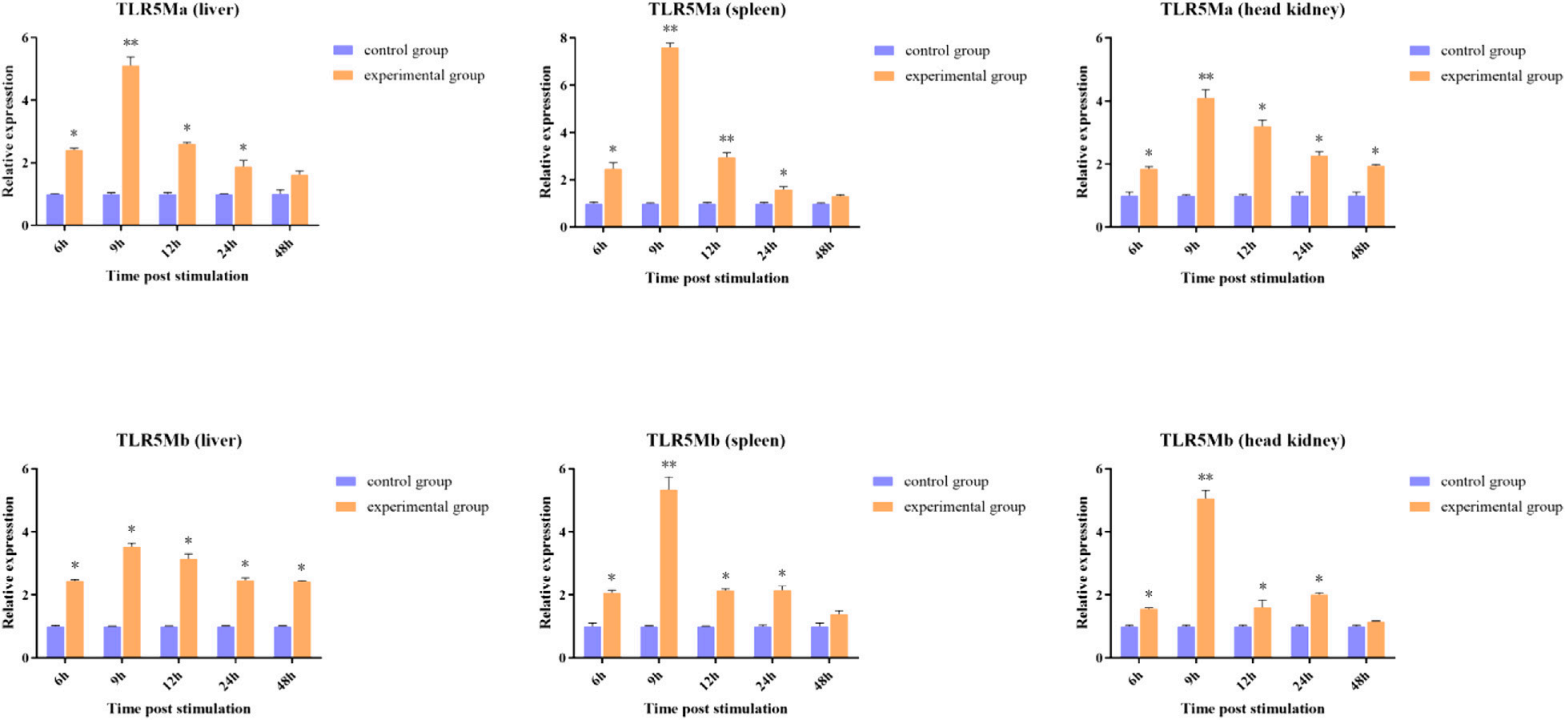
The expression patterns of *CiTLR5Ma* and *CiTLR5Mb* on transcription level in tissues of liver, spleen and head kidney, after infected with *A. hydrophila*. The expression level of *CiTLR5Ma* and *CiTLR5Mb* on 0 h was used as the baseline calibrator. The significance levels were obtained by comparing with corresponding 0 h (*p < 0.05, **p < 0.01).

To determine the role of *TLR5Ma* and *TLR5Mb* in the immune response against *A. hydrophila* infections, we examined their expression patterns in the liver, spleen, and head kidney tissues of *C. idellus* (Figure 4). The transcription levels of *CiTLR5a* and *CiTLR5b* were highest in the liver, spleen, and head kidney after 9 h of *A. hydrophila* infection. Subsequently, the transcription levels gradually declined but were still higher than the control group. These results suggest that *TLR5* plays a crucial role in the antibacterial immunity of *C. idellus*.

## 4 Discussion

### 4.1 Potential reasons for variation of TLR5 copy number among fishes

Two rounds of WGD have been proven to occur in the common ancestry of early vertebrates before ray-finned fishes–tetrapod split (Guyomard et al., 2012; Glasauer and Neuhauss, 2014). Specifically, the first round of WGD occurred before the split of agnatha–gnathostomata, and the second round occurred before the split of chrondrichthyes–osteichthyes. While the teleost-specific WGD, i.e., the third WGD, occurred solely in teleosts (Amores et al., 1998; Meyer and Schartl, 1999; Hoegg et al., 2004; Vandepoele et al., 2004). Moreover, a fourth WGD was observed in various teleost lineages, for instance, *Sinocyclocheilus* fishes, goldfish, salmonids, and common carp (Mayfield-Jones et al., 2013; Lien et al., 2016; Chen et al., 2019). Genome and gene duplications have contributed to teleosts’ rich evolutionary history and genomic diversity (Kaessmann, 2010).

**FIGURE 5:**
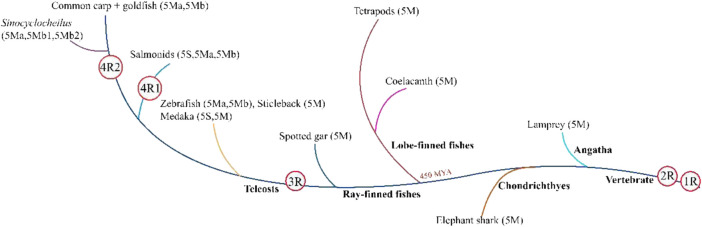
All teleost fishes undergone at least three whole-genome duplication (WGD) events, the two earlier rounds of vertebrate genome duplication (1R and 2R) that occurred before the di-vergence of lamprey and hagfish from the jawed vertebrates, and the third teleost-specific WGD (3R) at the base of the teleosts 320 million years ago (MYA). Atlantic salmon and river trout share the salmonid-specific WGD (4R 1) that occurred in the common ancestor of salmonids 80 MYA. Common carp and goldfish share the fourth WGD (4R) that occurred approximately 14 MYA in their common ancestor. Atlantic salmon and river trout share the salmonid-specific WGD (4R 1) that occurred in the common ancestor of salmonids 80 MYA. Common and goldfish share the fourth WGD (4R 2) that occurred approximately 14 MYA in their common ancestor. The *TLR5* isoform is given in parentheses.

According to the present study, WGD and gene loss may lead to copy number variations of the TLR5 gene in various fishes. Tetrapods and ancient fishes, including *Actinistia coelacanth* and *Neopterygii spotted-gar*, possess only one *TLR5* gene, i.e., *TLR5M*. The *TLR5* gene formed two copies, *TLR5M* and *TLR5S*, after undergoing teleost-specific WGD; therefore, two copies of the gene might be present in the teleost genomes compared to their tetrapod counterparts. Therefore, the teleost *TLR5* gene splits into two forms, including *TLR5M* and *TLR5S*. Furthermore, the existence of two *TLR5* genes is due to tandem duplication and not genome duplication (Figure 5).

In tetraploid teleosts, such as rainbow trout, the salmonid-specific genome duplication develops three copies of the *TLR5* genes, also studied in the Atlantic salmon (Lien et al., 2016). TLR5S was lost in other tetraploid carps, generating two TLR5M genes in its genome. However, in tetraploid golden-line fishes, three TLR5M isoforms are present, including TLR5Ma, TLR5Mb1, and TLR5Mb2. Additionally, as per the synteny analysis, the generation of three TLR5M genes is due to tandem (TLR5Ma and TLR5Mb1) and genome duplications (TLR5Mb2). Moreover, copy numbers are not always in the one-to-two correspondence between tetraploid and diploid species, presumably due to the selective loss of some copies.

### 4.2 Adaptive evolution of *TLR5* in fishes

Generally, WGD duplicates escape gene loss due to subfunctionalization or neofunctionalization (Jaillon et al., 2004). Teleost retained multiple isoforms, and Chondrichthyes only had one isoform of TLR5. The study revealed that fish TLR5 genes went through the primitive M (spotted gar), duplicated M/M (zebrafish), M/S (medaka and tilapia), tetraploid fish M/M/S (salmonids), and M/M/M (*Sinocyclocheilus* fish) evolution types. Membrane and soluble members of the M/S type play a synergistic role in sensing flagellin (Tsujita et al., 2004) possibly implying that soluble TLR5 is redundant in function and disappears in some fish and endothermic vertebrates. Cyprinid-specific duplicated membrane TLR5 (M/M type) demonstrated neofunctionalization to sense viral dsRNA as functional homodimeric receptors in antagonistic effect (Liao et al., 2022). Domain number variation was identified in the fish *TLR5* isotypes, generating functional diversity and complexity (Asami and Shimizu, 2021). Furthermore, the *TLR5* genes vary in the number of the extracellular LRR domain involved in the recognition of pathogens, contributing to *TLR5* specificity (Asami and Shimizu, 2021; Shan et al., 2021). These findings suggest that the duplicated membrane *TLR5* was sequence and species-specific for dsRNA binding. Many residues around the putative flagellin binding site were highly conserved among the TLR5 types, with only a few variations, such as residues 268 and 294. A study conducted previously indicates that these differences might influence the affinity of TLR5 for flagellins from different bacterial species (Andersen-Nissen et al., 2007). The fish TLR5 isoforms exhibited variations at the dsRNA binding sites (98 residues), possibly affecting viral immune response. Therefore, the newly predicated TLR5 duplicates in teleost may generate functional divergence and play a part in the adaption of these fishes during the evolutionary process.

Previous studies on *Cyprinus carpio* have revealed that TLR5M responds to flagellin from *A. hydrophila* (Duan et al., 2013). *TLR5M* transcription was subjected to remarkable upregulation after stimulation with lipopolysaccharide (LPS) in immune-related tissues in *Siniperca chuatsi* (Wang et al., 2021). The transcription of TLR5S and TLR5M was clearly altered after stimulation by polyinosinic:polycytidylic acid (poly (I:C)), LPS, and flagellin in immune-related organs of Golden Pompano, *Trachinotus ovatus* (Zhu et al., 2020). Moreover, the expression level of *TLR5* was remarkably upregulated in all tissues tested of Nile tilapia in response to *Streptococcus agalactiae* infection (Gao et al., 2022). These studies indicated that TLR5 plays important roles in the immune response to pathogen invasion. For the investigation of the expression of genes in response to *A. hydrophila* infection, to determine the role of *TLR5M* duplicates in the immune response, we employed Quantitative real-time Polymerase Chain Reaction (qRT-PCR). After *A. hydrophila* infection, overexpression of *CiTLR5Ma* and *CiTLR5Mb* genes were observed in immune system tissues and vital organs, including the liver, spleen, and head kidney, suggestive of their important roles in the immune response to the invasion of pathogens. Moreover, *CiTLR5Ma* and *CiTLR5Mb* showed similar expression patterns across tissues, which supports the finding that *CiTLR5Ma* and *CiTLR5Mb* are subfunctionalized to sense bacterial flagellin as a heterodimer (Liao et al., 2022).

## 5 Conclusion

In summary, the present study surveyed many facets of the fish *TLR5* genes, offering a worldwide genomic view of the diversity of the *TLR5* gene family of the fish. The existence of three *TLR5M* genes, *TLR5Ma*, *TLR5Mb1*, and *TLR5Mb2*, and one *TLR5S* in fish was confirmed in the study. Furthermore, the copy number variation in *TLR5* genes in fishes likely resulted from the integration of WGD and gene loss. In addition, differences between the flagellin interaction sites and viral binding sites were identified among *TLR5* isoforms, speculating that the fish *TLR5* genes possibly have different functions. The present study indicated that *TLR5* duplicates are expressed in several tissues with similar transcription levels after a bacterial infection. These findings supported the view that *TLR5* duplicates obtain sub-functionalization or neofunctionalization to sense pathogens.

## Data Availability

The datasets presented in this study can be found in online repositories. The names of the repository/repositories and accession number(s) can be found in the article/Supplementary Material.
