# Supplementary material for: Genome-wide identification and characterization of toll-like receptor 5 (TLR5) in fishes
Source: Front Genet. 2023 Jan 6;13:1083578. doi: 10.3389/fgene.2022.1083578 (PMC9857387; doi:10.3389/fgene.2022.1083578)
Supplement: Supplementary file 1 [file DataSheet1.PDF]

Table S1 List of primers used for qRT-qPCR.

Primers	Sequences (5’ - 3’)	Length (bp)	Accession	Purpose
Ci-βactin-F	TCTGCTATGTGGCTCTTGACTT	131	M25013	qRT-PCR
Ci-βactin-R	CTCTGGGCACCTGAACCTCT	131	M25013	qRT-PCR
Ci-TLR5Ma-F	TGTGGATGGAAAGCGAGAGG	113		qRT-PCR
Ci-TLR5Ma-R	CTAGCCTGTTCAGCAGTGCT	113		qRT-PCR
Ci-TLR5Mb-F	TGAGGACATTCTGGTGGTGC	133		qRT-PCR
Ci-TLR5Mb-R	CCAATCCAAGTCCTGGCTGT	133		qRT-PCR

Ci-TLR5Ma-F, Ci-TLR5Ma-R, Ci-TLR5Mb-F, Ci-TLR5Mb-R were designed by ourselves without NCBI Accession.
