# Supplementary material for: Genome-wide identification and characterization of toll-like receptor 5 (TLR5) in fishes
Source: Front Genet. 2023 Jan 6;13:1083578. doi: 10.3389/fgene.2022.1083578 (PMC9857387; doi:10.3389/fgene.2022.1083578)
Supplement: Supplementary file 2 [file Image1.PDF]

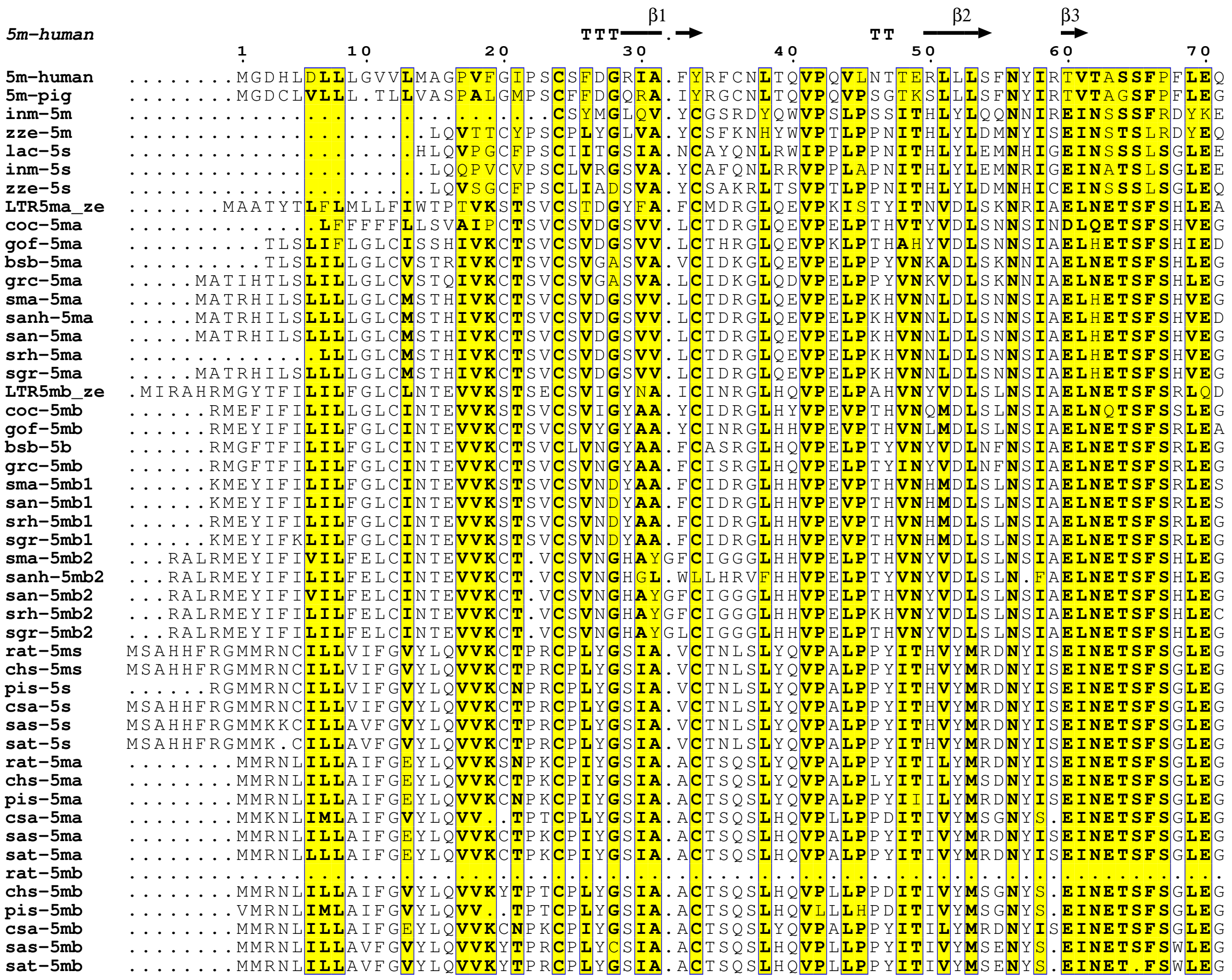
5m-human
β1 β2 β3
TTT TT
1 10 20 30 40 50 60 70
5m-human ........MGDHLDLLLGVVLMAGPVFGIPSCSFDGRIA.FYRFCNLTQVPQVLNTTERLLLSFNYIRTVTASSFPFLEQ
5m-pig ........MGDCLVLLL.TLLVASPALGMPSCFFDGQRA.IYRGCNLTQVPQVPSGTKSLLLSFNYIRTVTAGSFPFLEG
inm-5m ....CSYMGLQV.YCGSRDYQWVPSLPSSITHLYLQQNNIREINSSSFRDYKE
zze-5m ......................LQVTTCYPSCPLYGLVA.YCSFKNHYWVPTLPPNITHLYLDMNYISEINSTSLRDYEQ
lac-5s .....................HLQVPGCFPSCIITGSIA.NCAYQNLRWIPPLPPNITHLYLEMNHIGEINSSSLSGLEE
inm-5s ......................LQQPVCVPSCLVRGSVA.YCAFQNLRRVPPLAPNITHLYLEMNRIGEINATSLSGLEE
zze-5s ......................LQVSGCFPSCLIADSVA.YCSAKRLTSVPTLPPNITHLYLDMNHICEINSSSLSGLEQ
LTR5ma_ze .......MAATYTLFLMLLFIWTPTVKSTSVCSTDGYFA.FCMDRGLQEVPKISTYITNVDLSKNRIAELNETSFSHLEA
coc-5ma ..............LFFFFFLLSVAIPCTSVCSVDGSVV.LCTDRGLQEVPELPTHVTYVDLSNNSINDLQETSFSHVEG
gof-5ma ..........TLSLIFLGLCISSHIVKCTSVCSVDGSVV.LCTHRGLQEVPKLPTHAHYVDLSNNSIAELHETSFSHIED
bsb-5ma ..........TLSLILLGLCVSTRIVKCTSVCSVGASVA.VCIDKGLQEVPELPPYVNKADLSKNNIAELNETSFSHLEG
grc-5ma .....MATIHTLSLILLGLCVSTQIVKCTSVCSVGASVA.LCIDKGLQDVPELPPYVNKVDLSKNNIAELNETSFSHLEG
sma-5ma .....MATRHILSLLLLGLCMSTHIVKCTSVCSVDGSVV.LCTDRGLQEVPELPKHVNNLDLSNNSIAELHETSFSHVEG
sanh-5ma .....MATRHILSLLLLGLCMSTHIVKCTSVCSVDGSVV.LCTDRGLQEVPELPKHVNNLDLSNNSIAELHETSFSHVED
san-5ma .....MATRHILSLLLLGLCMSTHIVKCTSVCSVDGSVV.LCTDRGLQEVPELPKHVNNLDLSNNSIAELHETSFSHVEG
srh-5ma ..............LLLGLCMSTHIVKCTSVCSVDGSVV.LCTDRGLQEVPELPKHVNNLDLSNNSIAELHETSFSHVEG
sgr-5ma .....MATRHILSLLLLGLCMSTHIVKCTSVCSVDGSVV.LCIDRGLQEVPELPKHVNNLDLSNNSIAELHETSFSHVEG
LTR5mb_ze .MIRAHRMGYTFILILFGLCLNTEVVKSTSECSVIGYNA.ICINRGLHQVPELPAHVNYVDLSLNSIAELNETSFSRLQD
coc-5mb ......RMEFIFILILLGLCINTEVVKSTSVCSVIGYAA.YCIDRGLHYVPEVPTHVNQMDLSLNSIAELNQTSFSSLEG
gof-5mb ......RMEYIFILILFGLCINTEVVKCTSVCSVYGYAA.YCINRGLHHVPEVPTHVNLMDLSLNSIAELNETSFSRLEA
bsb-5b ......RMGFTFILILFGLCINTEVVKCTSVCLVNGYAA.FCASRGLHQVPELPTYVNYVDLNFNSIAELNETSFSRLEG
grc-5mb ......RMGFTFILILFGLCINTEVVKCTSVCSVNGYAA.FCISRGLHQVPELPTYINYVDLNFNSIAELNETSFSRLEG
sma-5mb1 ......KMEYIFILILFGLCINTEVVKSTSVCSVNDYAA.FCIDRGLHHVPEVPTHVNHMDLSLNSIAELNETSFSRLES
san-5mb1 ......KMEYIFILILFGLCINTEVVKSTSVCSVNDYAA.FCIDRGLHHVPEVPTHVNHMDLSLNSIAELNETSFSRLES
srh-5mb1 ......RMEYIFILILFGLCINTEVVKSTSVCSVNDYAA.FCIDRGLHHVPEVPTHVNHMDLSLNSIAELNETSFSRLEG
sgr-5mb1 ......KMEYIFKLILFGLCINTEVVKSTSVCSVNDYAA.FCIDRGLHHVPEVPTHVNHMDLSLNSIAELNETSFSRLEG
sma-5mb2 ...RALRMEYIFIVILFELCINTEVVKCT.VCSVNGHAYGFCIGGGLHHVPELPTHVNYVDLSLNSIAELNETSFSHLEG
sanh-5mb2 ...RALRMEYIFILILFELCINTEVVKCT.VCSVNGHGL.WLLHRVFHHVPELPTYVNYVDLSLN.FAELNETSFSHLEG
san-5mb2 ...RALRMEYIFIVILFELCINTEVVKCT.VCSVNGHAYGFCIGGGLHHVPELPTHVNYVDLSLNSIAELNETSFSHLEG
srh-5mb2 ...RALRMEYIFILILFELCINTEVVKCT.VCSVNGHAYGFCIGGGLHHVPELPKHVNYVDLSLNSIAELNETSFSHLEC
sgr-5mb2 ...RALRMEYIFILILFELCINTEVVKCT.VCSVNGHAYGLCIGGGLHHVPELPTHVNYVDLSLNSIAELNETSFSHLEG
rat-5ms MSAHHFRGMMRNCILLVIFGVYLQVVKCTPRCPLYGSIA.VCTNLSLYQVPALPPYITHVYMRDNYISEINETSFSGLEG
chs-5ms MSAHHFRGMMRNCILLVIFGVYLQVVKCTPRCPLYGSIA.VCTNLSLYQVPALPPYITHVYMRDNYISEINETSFSGLEG
pis-5s ......RGMMRNCILLVIFGVYLQVVKCNPRCPLYGSIA.VCTNLSLYQVPALPPYITHVYMRDNYISEINETSFSGLEG
csa-5s MSAHHFRGMMRNCILLVIFGVYLQVVKCTPRCPLYGSIA.VCTNLSLYQVPALPPYITHVYMRDNYISEINETSFSGLEG
sas-5s MSAHHFRGMMKKCILLAVFGVYLQVVKCTPRCPLYGSIA.VCTNLSLYQVPALPPYITHVYMRDNYISEINETSFSGLEG
sat-5s MSAHHFRGMMK.CILLAVFGVYLQVVKCTPRCPLYGSIA.VCTNLSLYQVPALPPYITHVYMRDNYISEINETSFSGLEG
rat-5ma ........MMRNLILLAIFGEYLQVVKSNPKCPIYGSIA.ACTSQSLYQVPALPPYITILYMRDNYISEINETSFSGLEG
chs-5ma ........MMRNLILLAIFGEYLQVVKCTPKCPIYGSIA.ACTSQSLYQVPALPLYITILYMSDNYISEINETSFSGLEG
pis-5ma ........MMRNLILLAIFGEYLQVVKCNPKCPIYGSIA.ACTSQSLYQVPALPPYIIILYMRDNYISEINETSFSGLEG
csa-5ma ........MMKNLIMLAIFGVYLQVV..TPTCPLYGSIA.ACTSQSLHQVPLLPPDITIVYMSGNYS.EINETSFSGLEG
sas-5ma ........MMRNLILLAIFGEYLQVVKCTPKCPIYGSIA.ACTSQSLHQVPALPPYITIVYMRDNYISEINETSFSGLEG
sat-5ma ........MMRNLLLLAIFGEYLQVVKCTPKCPIYGSIA.ACTSQSLHQVPALPPYITIVYMRDNYISEINETSFSGLEG
rat-5mb ....
chs-5mb ........MMRNLILLAIFGVYLQVVKYTPTCPLYGSIA.ACTSQSLHQVPLLPPDITIVYMSGNYS.EINETSFSGLEG
pis-5mb ........VMRNLIMLAIFGVYLQVV..TPTCPLYGSIA.ACTSQSLHQVLLLHPDITIVYMSGNYS.EINETSFSGLEG
csa-5mb ........MMRNLILLAIFGEYLQVVKCNPKCPIYGSIA.ACTSQSLYQVPALPPYITILYMRDNYISEINETSFSGLEG
sas-5mb ........MMRNLILLAVFGVYLQVVKYTPRCPLYCSIA.ACTSQSLHQVPLLPPYITIVYMSENYS.EINETSFSWLEG
sat-5mb ........MMRNLILLAVFGVYLQVVKYTPRCPLYGSIA.ACTSQSLHQVPLLPPYITIVYMSENYS.EINET.FSWLEG

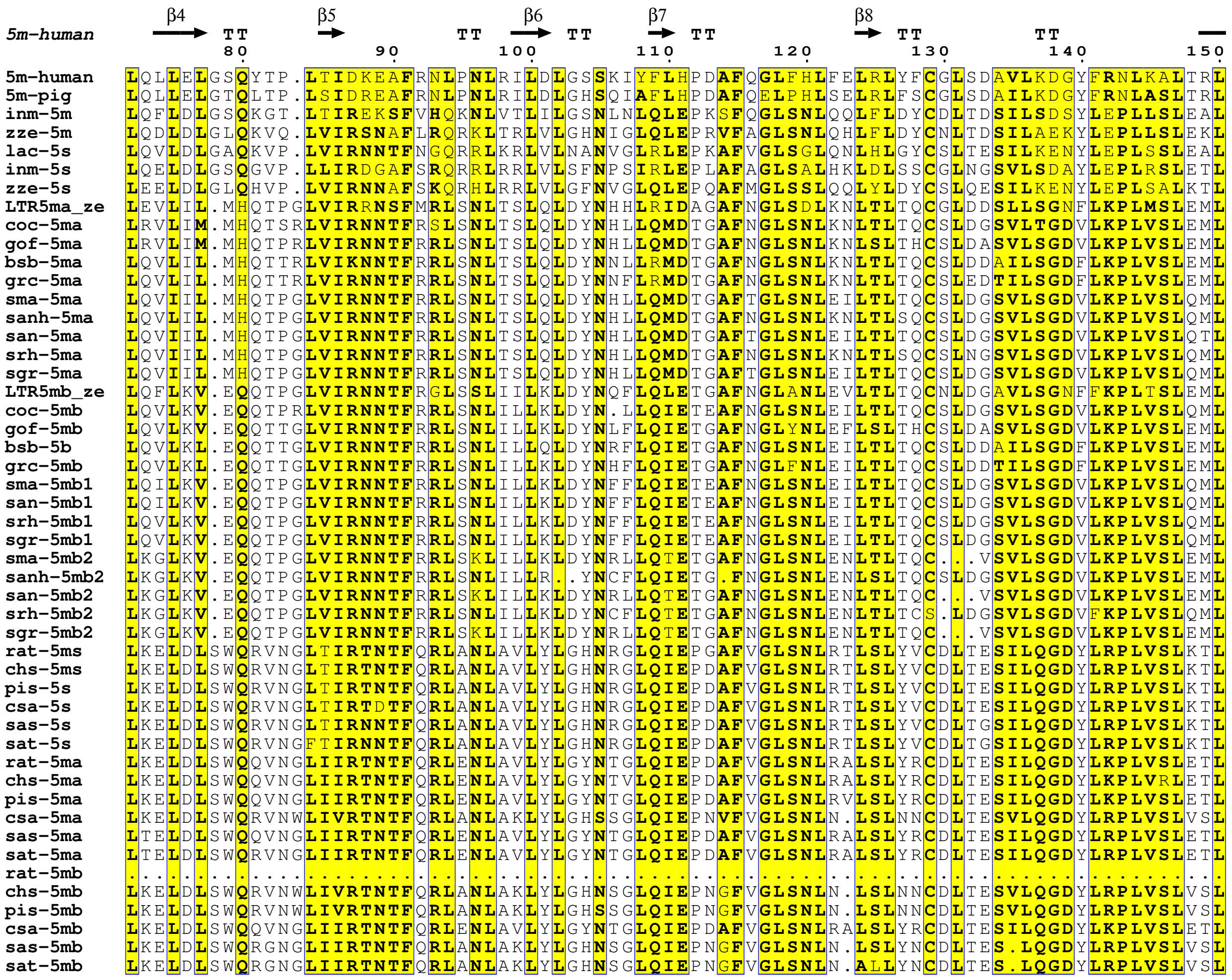
β4 β5 β6 β7 β8
5m-human TT TT TT TT TT TT
80 90 100 110 120 130 140 150
5m-human LQLELGSQYTP.LTIDKEAFRNLPNLRILDLGSSKIYFLHPDAFQGLFHLFELRLYFCGLSDAVLKDGYFRNLKALTRL
5m-pig LQLELGTQLTP.LSIDREAFRNLPNLRILDLGHSQIAFLHPDAFQELPHLSELRLFSCGLSDAILKDGYFRNLASLTRL
inm-5m LQFLDLGSQKGT.LTIREKSFVHQKNLVTLILGSNLNLQLEPKSFQGLSNLQQLFLDYCDLTDSILSDSYLEPLLSLEAL
zze-5m LQDLDLGLQKVQ.LVIRSNAFLRQRKLTRLVLGHNIGLQLEPRVFAGLSNLQHLFLDYCNLTDSILAEKYLEPLLSLEKL
lac-5s LQVLDLGAQKVP.LVIRNNTFNGQRRLKRLVLNANVGLRLEPKAFVGLSGLQNLHLGYCSLTESILKENYLEPLSSLEAL
inm-5s LQELDLGSQGVP.LLIRDGAFSRQRRLRRLVLSFNPSIRLEPLAFAGLSALHKLDLSSCGLNGSVLSDAYLEPLRSLETL
zze-5s LEELDLGLQHVP.LVIRNNAFSKQRHLRRLVLGFNVGLQLEPQAFMGLSSLQQLYLDYCSLQESILKENYLEPLSALKTL
LTR5ma_ze LEVLIL.MHQTPGLVIRRNSFMRLSNLTSLQLDYNHHLRIDAGAFNGLSDLKNLTLTQCGLDDSLLSGNFLKPLMSLEML
coc-5ma LRVLIM.MHQTSRLVIRNNTFRSLSNLTSLQLDYNHLLQMDTGAFNGLSNLKNLTLTQCSLDGSVLTGDVLKPLVSLEML
gof-5ma LRVLIM.MHQTPRLVIRNNTFRRLSNLTSLQLDYNHLLQMDTGAFNGLSNLKNLSLTHCSLDASVLSGDVLKPLVSLEML
bsb-5ma LQVLIL.MHQTTRLVIKNNTFRRLSNLTSLQLDYNNLLRMDTGAFNGLSNLKNLTLTQCSLDDAILSGDFLKPLVSLEML
grc-5ma LQVLIL.MHQTTRLVIRNNTFRRLSNLTSLQLDYNNFLRMDTGAFNGLSNLKNLTLTQCSLEDTILSGDFLKPLVSLEML
sma-5ma LQVIIL.MHQTPGLVIRNNTFRRLSNLTSLQLDYNHLLQMDTGAFTGLSNLEILTLTQCSLDGSVLSGDVLKPLVSLQML
sanh-5ma LQVLIL.MHQTPGLVIRNNTFRRLSNLTSLQLDYNHLLQMDTGAFNGLSNLKNLTLSQCSLDGSVLSGDVLKPLVSLQML
san-5ma LQVIIL.MHQTPGLVIRNNTFRRLSNLTSLQLDYNHLLQMDTGAFTGLSNLEILTLTQCSLDGSVLSGDVLKPLVSLQTL
srh-5ma LQVIIL.MHQTPGLVIRNNTFRRLSNLTSLQLDYNHLLQMDTGAFNGLSNLKNLTLSQCSLNGSVLSGDVLKPLVSLQML
sgr-5ma LQVIIL.MHQTPGLVIRNNTFRRLSNLTSLQLDYNHLLQMDTGAFTGLSNLEILTLTQCSLDGSVLSGDVLKPLVSLQML
LTR5mb_ze LQFLKV.EQQTPGLVIRNNTFRGLSSLIILKLDYNQFLQLETGAFNGLANLEVLTLTQCNLDGAVLSGNFFKPLTSLEML
coc-5mb LQVLKV.EQQTPRLVIRNNTFRRLSNLILLKLDYN.LLQIETEAFNGLSNLEILTLTQCSLDGSVLSGDVLKPLVSLQML
gof-5mb LQVLKV.EQQTTGLVIRNNTFRRLSNLILLKLDYNLFLQIETGAFNGLYNLEFLSLTHCSLDASVLSGDVLKPLVSLEML
bsb-5b LQVLKL.EQQTTGLVIRNNTFRRLSNLILLQLDYNRFLQIETGAFNGLSNLEILTLTQCSLDDAILSGDFLKPLVSLEML
grc-5mb LQVLKL.EQQTTGLVIRNNTFRRLSNLILLKLDYNHFLQIETGAFNGLFNLEILTLTQCSLDDTILSGDFLKPLVSLEML
sma-5mb1 LQILKV.EQQTPGLVIRNNTFRRLSNLILLKLDYNFFLQIETEAFNGLSNLEILTLTQCSLDGSVLSGDVLKPLVSLQML
san-5mb1 LQILKV.EQQTPGLVIRNNTFRRLSNLILLKLDYNFFLQIETEAFNGLSNLEILTLTQCSLDGSVLSGDVLKPLVSLQML
srh-5mb1 LQVLKV.EQQTPGLVIRNNTFRRLSNLILLKLDYNFFLQIETEAFNGLSNLEILTLTQCSLDGSVLSGDVLKPLVSLQML
sgr-5mb1 LQVLKV.EQQTPGLVIRNNTFRRLSNLILLKLDYNFFLQIETEAFNGLSNLEILTLTQCSLDGSVLSGDVLKPLVSLQML
sma-5mb2 LKGLKV.EQQTPGLVIRNNTFRRLSKLILLKLDYNRLLQTETGAFNGLSNLENLTLTQC...VSVLSGDVLKPLVSLEML
sanh-5mb2 LKGLKV.EQQTPGLVIRNNTFRRLSNLILLR..YNCFLQIETG.FNGLSNLENLSLTQCSLDGSVLSGDVLKPLVSLEML
san-5mb2 LKGLKV.EQQTPGLVIRNNTFRRLSKLILLKLDYNRLLQTETGAFNGLSNLENLTLTQC...VSVLSGDVLKPLVSLEML
srh-5mb2 LKGLKV.EQQTPGLVIRNNTFRRLSNLILLKLDYNCFLQTETGAFNGLSNLENLTLTCS.LDGSVLSGDVFKPLVSLQML
sgr-5mb2 LKGLKV.EQQTPGLVIRNNTFRRLSKLILLKLDYNRLLQTETGAFNGLSNLENLTLTQC...VSVLSGDVLKPLVSLEML
rat-5ms LKELDLSWQRVNGLTIRTNTFQRLANLAVLYLGHNRGLQIEPGAFVGLSNLRTLSLYVCDLTESILQGDYLRPLVSLKTL
chs-5ms LKELDLSWQRVNGLTIRTNTFQRLANLAVLYLGHNRGLQIEPDAFVGLSNLRTLSLYVCDLTESILQGDYLRPLVSLKTL
pis-5s LKELDLSWQRVNGLTIRTNTFQRLANLAVLYLGHNRGLQIEPDAFVGLSNLRTLSLYVCDLTESILQGDYLRPLVSLKTL
csa-5s LKELDLSWQRVNGLTIRTDTFQRLANLAVLYLGHNRGLQIEPDAFVGLSNLRTLSLYVCDLTESILQGDYLRPLVSLKTL
sas-5s LKELDLSWQRVNGLTIRNNTFQRLANLAVLYLGHNRGLQIEPDAFVGLSNLRTLSLYVCDLTGSILQGDYLRPLVSLKTL
sat-5s LKELDLSWQRVNGFTIRNNTFQRLANLAVLYLGHNRGLQIEPDAFVGLSNLRTLSLYVCDLTGSILQGDYLRPLVSLKTL
rat-5ma LKELDLSWQQVNGLIIRTNTFQRLENLAVLYLGYNTGLQIEPDAFVGLSNLRALSLYRCDLTESILQGDYLKPLVSLETL
chs-5ma LKELDLSWQQVNGLIIRTNTFQRLENLAVLYLGYNTVLQIEPDAFVGLSNLRALSLYRCDLTESILQGDYLKPLVRLETL
pis-5ma LKELDLSWQQVNGLIIRTNTFQRLENLAVLYLGYNTGLQIEPDAFVGLSNLRVLSLYRCDLTESILQGDYLKPLVSLETL
csa-5ma LKELDLSWQRVNWLIVRTNTFQRLANLAKLYLGHSSGLQIEPNVFVGLSNLN.LSLNNCDLTESVLQGDYLRPLVSLVSL
sas-5ma LTELDLSWQQVNGLIIRTNTFQRLENLAVLYLGYNTGLQIEPDAFVGLSNLRALSLYRCDLTESILQGDYLRPLVSLETL
sat-5ma LTELDLSWQRVNGLIIRTNTFQRLENLAVLYLGYNTGLQIEPDAFVGLSNLRALSLYRCDLTESILQGDYLRPLVSLETL
rat-5mb ....
chs-5mb LKELDLSWQRVNWLIVRTNTFQRLANLAKLYLGHNSGLQIEPNGFVGLSNLN.LSLNNCDLTESVLQGDYLRPLVSLVSL
pis-5mb LKELDLSWQRVNWLIVRTNTFQRLANLAKLYLGHSSGLQIEPNGFVGLSNLN.LSLNNCDLTESVLQGDYLRPLVSLVSL
csa-5mb LKELDLSWQQVNGLIIRTNTFQRLENLAVLYLGYNTGLQIEPDAFVGLSNLRALSLYRCDLTESILQGDYLKPLVSLETL
sas-5mb LKELDLSWQRGNGLIIRTNTFQRLANLAKLYLGHNSGLQIEPNGFVGLSNLN.LSLYNCDLTES.LQGDYLRPLVSLVSL
sat-5mb LKELDLSWQRGNGLIIRTNTFQRLANLAKLYLGHNSGLQIEPNGFVGLSNLN.ALLYNCDLTES.LQGDYLRPLVSLVSL

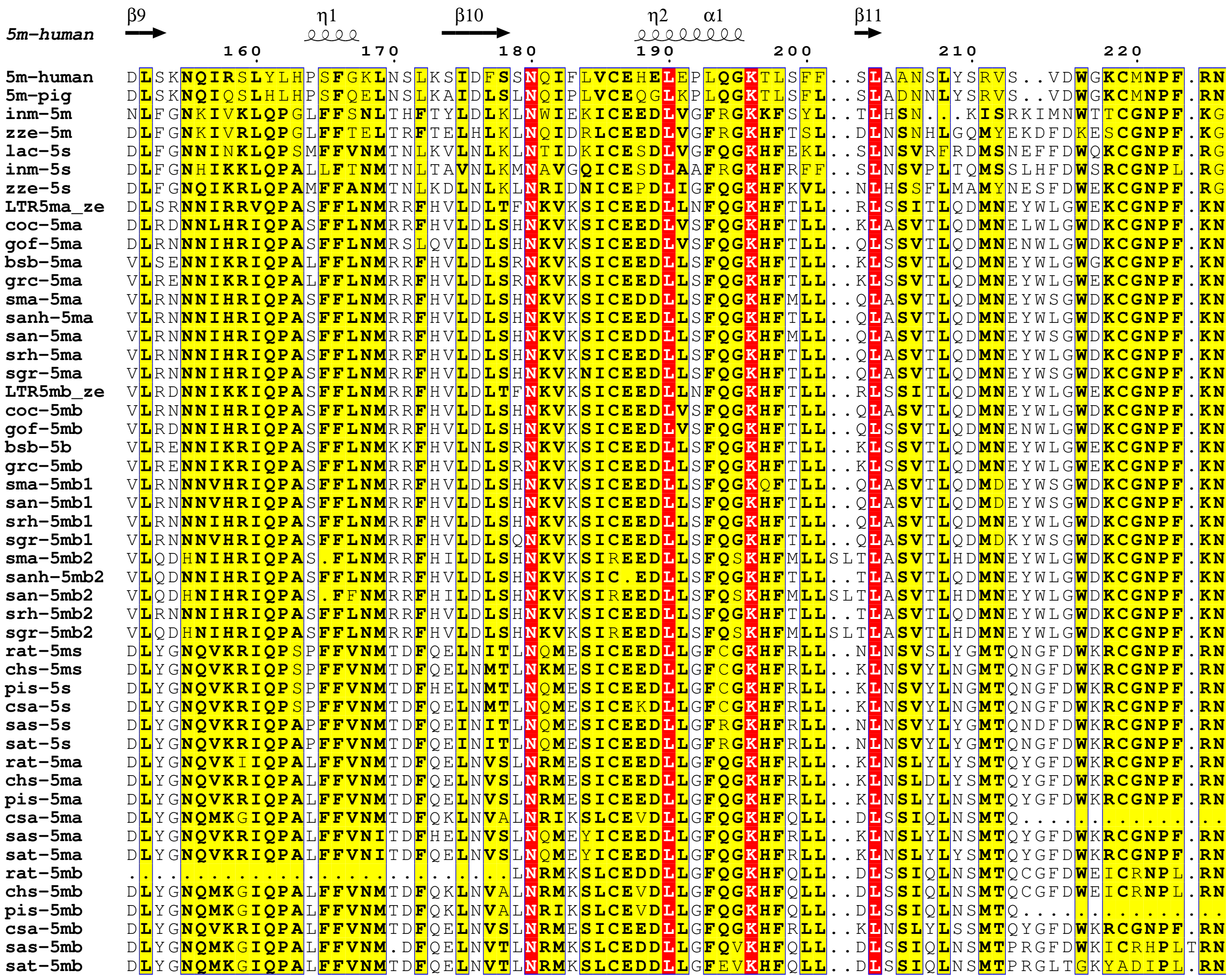

5m-human
β9
η1
β10
η2
α1
β11
160
170
180
190
200
210
220
5m-human
5m-pig
inm-5m
zze-5m
lac-5s
inm-5s
zze-5s
LTR5ma_ze
coc-5ma
gof-5ma
bsb-5ma
grc-5ma
sma-5ma
sanh-5ma
san-5ma
srh-5ma
sgr-5ma
LTR5mb_ze
coc-5mb
gof-5mb
bsb-5b
grc-5mb
sma-5mb1
san-5mb1
srh-5mb1
sgr-5mb1
sma-5mb2
sanh-5mb2
san-5mb2
srh-5mb2
sgr-5mb2
rat-5ms
chs-5ms
pis-5s
csa-5s
sas-5s
sat-5s
rat-5ma
chs-5ma
pis-5ma
csa-5ma
sas-5ma
sat-5ma
rat-5mb
chs-5mb
pis-5mb
csa-5mb
sas-5mb
sat-5mb

```
              β12            η3         β13                     η4            β14 TT   β15
5m-human
              230       240       250       260       270       280       290       300
5m-human      MVLEILDVSGNGWTVDITGNFSNAISKSQAFSLILAHHI.MGAGFGFHNIKDPDQNTFAGLARSSVRHLDLSHGFVFSLN
5m-pig        MALEMLDVSGNGWTADTTRNFSQAVNGSQISSLVLAHHI.MGSGFGFHNIKDPDHHTFASLGRSSLIQLDLSHGFIFSLN
inm-5m        VSFGTLDLSSNGFDLEKTKQFFQAIRGTQIDRLIYS.GG.LGKGFSFNNFLDPNESTFQGLQDSSLAIFEIAGNRIFALL
zze-5m        LTFNILELSSNGFSINTAIQFFRAIEGTQIAHLIFS.GH.MGKGFSHDNLPDPDEHTFAGLMNSAVNILDLSQNSIFALK
lac-5s        ISFQSLDLSNNGLSLGKSKQFFRAIKGTKISHLKMS.GH.IGKGFSFNNLPDPDRSTFEGLTNSSILTLDLSKNRIFALQ
inm-5s        MSFQDLDLSNSMLGAQKLKLLFRALEGTKISHLKLS.GH.MGRGFSFGNLPDPDHSTFEGLHNSSVLSLSLSKNRIFALQ
zze-5s        MSFQTLDLSSNGFSVGTLKQFCRAIEGAKISHLILS.GS.MGKGFSFSNFPDPDHSTFEGLKKSSVHILDLSKNRIFALQ
LTR5ma_ze     TSITTLDLSGNGFNVDMAKRFFDAISSTKIQSLIISNTYRIGKSSG.DNSKDPDKSTFSGLKSSGIKSFDLSNSSIFSLT
coc-5ma       MSINVLDLSGN.FNVNTAKRFFNAISATKIQSLIFSNICSLGRSSG.NNSKDPDKFTFSGLEASGIKTFDLSSSNIFSLP
gof-5ma       MSVTELDLSGNGFNVNIAKRFFNAIRATKIQSLIFSNICSLGRSSG.NNSQDPDRFTFNGLEASGIKTFDLSSSNIFSLS
bsb-5ma       MSITILDLSGNGFNVNMAKRYFDAITGTKIQSLILSNSYSMGRSFG.NNSKDPDKFTFKGLEASAITIFDLSNSSIFALS
grc-5ma       MSVSVLDLSGNGFNDNNAKLFFDAITGTKIQSLILSNSHSMGSSSG.NNSKDPNKFTFKGLEASGIKIFDLSNSSIFALS
sma-5ma       MSVTVLDLSGNGFNVNIAKRFFNAISGTKIQSLIFSNICSLGRSSG.NNSKDPDKFTFKGLEASGVKTFDLSSSNIFSLS
sanh-5ma      MSVTVLDLSGNGFNVNIAKRFFNAVSGTKIQSLIFSNICSLGRSSG.NNSKDPDKFTFKGLEASGVKTFDLSSSNIFSLS
san-5ma       MSVTVLDLSGNGFNVNIAKRFFNAISGTKIQSLIFSNICSLGRSSG.NNSKDPDKFTFKGLEASGVKTFDLSSSNIFSLS
srh-5ma       MSVTVLDLSGNGFKVPMAKRFFDAITGTKILGLILSNSYNMGSSFGHNNFPDPDKFTFKGLEASGVKTFDLSSSNIFSLS
sgr-5ma       MSVTVLDLSGNGFNVNIAKRFFNAISGTKIQSLIFSNICSLGRSSG.NNSKDPDKFTFKGLEASGVKTFNLSSSNIFSLS
LTR5mb_ze     TSITTLDLSGNGFKESMAKRFFDAIAGTKIQSLILSNSYNMGSSFGHTNFKDPDNFTFKGLEASGVKTCDLSKSKIFALL
coc-5mb       MSVTVLDLSGNGFQVPMAKRFFDAITGTKILGLILSKSYSMGSSFSHNNFPDPDKFTFKGLEVSGVKIFDLSQSNIFALS
gof-5mb       MSVNVLDLSGNGFNVPMAKRFFDAFTSTKILGLILSKSYNMGSSFGHNNFPDPDKFTFKGLEVSGVKVFDLSQSHIFALS
bsb-5b        MSITILDLSGNGFNVNMAKRFFDAITGTKIQSLILSNSYSMGSSFSYNNFKDPDKFTFKGLGDSGIEIFDLSKSKIFALS
grc-5mb       MSVSVLDLSGNGFNVKMAKLFFDAITGTKIQSLILSNSYSMGSSFGHNNFKDPDKFTLKGLGDSGIKIFDLSKSQIFALS
sma-5mb1      MSITVLDLSGNGFKVPMAKRFFDAITGTKILGLILSNSYNMGSSFGHNNFPDPDKFTFKGLEASGVKVFDLSQSHIFALS
san-5mb1      MSITVLDLSGNGFKVPMAKRFFDAITGTKILGLILSNSYNMGSSFGHNNFPDPDKFTFKGLEASGVKVFDLSQSHIFALS
srh-5mb1      MSVTVLDLSGNGFKVPMAKRFFDAITGTKILGLILSNSYNMGSSFGHNNFPDPDKFTFKGLEVSGLKVFDLSQSHIFALS
sgr-5mb1      MSITVLDLSGNGFKVPMAKRFFDAITGTKILGLILSNSYNMGSSFGHNNFPDPDKFTFKGLEASGVKVFDLSQSHIFALS
sma-5mb2      MSIMVLDLSGNSFKVPMAKRFFLCNH...YLRLILSKSYNIGSSFGHNNFPDPDKFTFKRLEQGCPNSGDLSQSHIFALS
sanh-5mb2     MSVMVLDLSGNGFKVPMAKRFFDAFTGPEIQGLILSRSYNIGSSFAPNNFPDPDKFTFKGLEVSGVKIFDLSQSHIFTLS
san-5mb2      MSIMVLDLSGNSFKVPMAKRFFLCNH...YLRLILSKSYNIGSSFGHNNFPDPDKFTFKRLEQG.......AQTRSWRAS
srh-5mb2      MSVMVLDLSGNGFKVPMTKRFFDAII....IGLILSKSYNIGSSFGHNNFPDPDKFTFKGLEVSGVKIFDLSQSHIFALS
sgr-5mb2      MSIMVLDLSGNSFKVPMAKRFFFMQS...LFKLILSKSYNIGSSFGHNNFPDPDKFTFKRLEQGCPNSGDLSQSHIFALS
rat-5ms       MSIETLDLSSNGFNVDKAKLFFNAIQGTKIHHIILEHST.MGKSFGFSNFKDPNKKTFNGLKNSGIKILDLSKCFIFALQ
chs-5ms       MSIETLDLSSNGFNVDKAKLFFNAIQGTKIHHIILEHST.MGKSFGFNNFKDPNKKTFNGLKNSGIKILDLSKCFIFALQ
pis-5s        MSIETLDLSSNGFNVDKAKLFFNAIQGTKIHHIILEHST.MGKSFGFSNFKDPNKKTFNGLKNSGIKILNLSKCFIFALQ
csa-5s        MSIETLDLSSNGFNVDKAKLFFNAIQGTKIHHIILEHST.MGKSFGFSHFKDPNKKTFNGLKNSGIKILNLSKCFIFALQ
sas-5s        MSIETLDLSSNGFNVDKAKLFFNAIQGMKIHHIILKHST.MGKSFGFSNVKDPNKKTFNGLKNSGIKILDFSKCFIFALQ
sat-5s        MSIETLDLSSNGFNVDKAKLFFNAIQGTKIHHIILKHST.MGKSFGFSNVKDPNKKTFNGLKNSGIKILDFSKCFIFALQ
rat-5ma       MSMETLDLSSNGFDVDKAKLFFNAIQGTKIHHLILEHST.MGKSFGFSNVKDPDRQTFEGLENSSVKILDLSKCFIFTLQ
chs-5ma       MSMETLDLSNNGFDVDKAKLFFHAIQGTKIHHLILEHST.MGKSFGFSNVKDPDRQTFEGLKNSSVKILDLSKCFIFTLQ
pis-5ma       MSMETLDLSSNGFDVDKAKLFFNAIQGTKIHHLILEHST.MGKSFGFSNVKDPDRQTFEGLKSSSVKILDLSKCFIFTLQ
csa-5ma       ................................................................................
sas-5ma       MSMETLDLSSNGFDVDKAKLFFNAIRGTKIHHLILEHST.MGKSFGFSNVKDPDRQTFEGLKNSSVKILDLSKCFIFTLQ
sat-5ma       MSMETLDLSSNGFDVDKAKLFFNAIRGTKIHHLILEHST.MGKSFGFSNVKDPDRQTFEGLKNSSVKILDLSKCFIFTLQ
rat-5mb       MSMEILDLPSNGFNVDKAKLFFSAIRGTKIPHLILEHST.MGKSFCFSDVKDPDRKT.EGLKNSGVKILDLSKCFIFTLQ
chs-5mb       MSMEILDLPSNGFNVDKAKLFFSAIRGTKTPHLILEHST.MGKSFCFSDVKDPDRKT.EGFKNSGVKILDLSKCFIFTLQ
pis-5mb       ................................................................................
csa-5mb       MSMETLDLSSNGFDVDKAKLFFNAIQGTKIHHLILEHST.MGKSFGFSNVKDPDRQTFEGLKSSSVKILDLSKCFIFTLQ
sas-5mb       MSMEILDLPSNGFNVDKAKQFFSAIRGTKIPHLILEHST.MGK.FCFSNVKDPDRQT.EGFKNSGVKILDLSKCFIFTLQ
sat-5mb       MSMEILDLPSNGFNVDKAKLFFSAIG.TKIPHLILEHST.MGKSFCFSNVKDPDRQT.EGFKNSGVKILDLSKCFIFTLQ
```

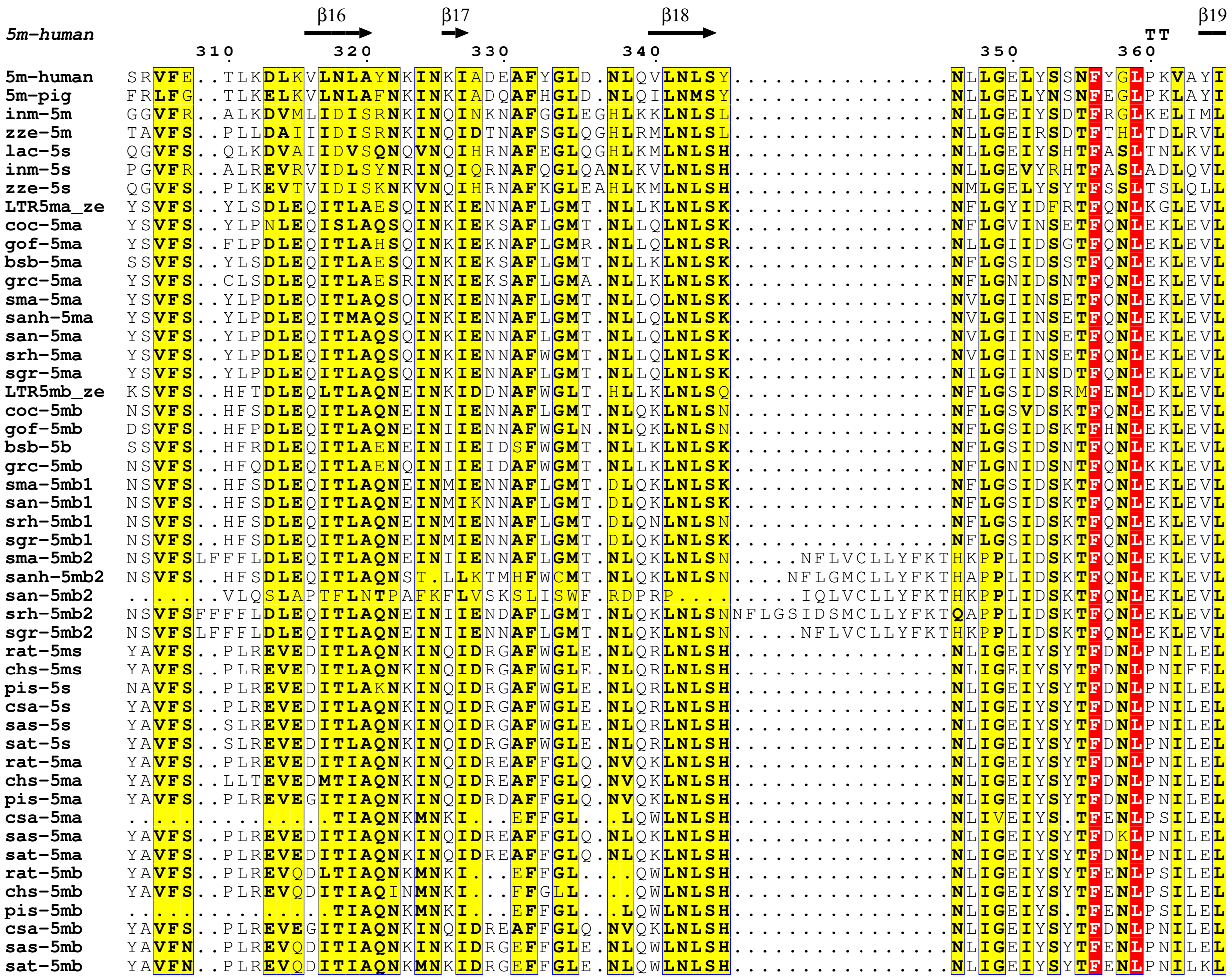

5m-human
β16 β17 β18 TT β19
310 320 330 340 350 360
5m-human SRVFE..TLKDLKVLNLAYNKINKIADEAFYGLD.NLQVLNLSY...............NLLGELYSSNFYGLPKVAYI
5m-pig FRLFG..TLKELKVLNLAFNKINKIADQAFHGLD.NLQILNMSY...............NLLGELYNSNFEGLPKLAYI
inm-5m GGVFR..ALKDVMLIDISRNKINQINKNAFGGLEGHLKKLNLSL...............NLLGEIYSDTFRGLKELIML
zze-5m TAVFS..PLLDAIIIDISRNKINQIDTNAFSGLQGHLRMLNLSL...............NLLGEIRSDTFTHLTDLRVL
lac-5s QGVFS..QLKDVAIIDVSQNQVNQIHRNAFEGLQGHLKMLNLSH...............NLLGEIYSHTFASLTNLKVL
inm-5s PGVFR..ALREVRVIDLSYNRINQIQRNAFQGLQANLKVLNLSH...............NLLGEVYRHTFASLADLQVL
zze-5s QGVFS..PLKEVTVIDISKNKVNQIHRNAFKGLEAHLKMLNLSH...............NMLGELYSYTFSSLTSLQLL
LTR5ma_ze YSVFS..YLSDLEQITLAESQINKIENNAFLGMT.NLLKLNLSK...............NFLGYIDFRTFQNLKGLEVL
coc-5ma YSVFS..YLPNLEQISLAQSQINKIEKSAFLGMT.NLLQLNLSK...............NFLGVINSETFQNLEKLEVL
gof-5ma YSVFS..FLPDLEQITLAHSQINKIEKNAFLGMR.NLLQLNLSR...............NLLGIIDSGTFQNLEKLEVL
bsb-5ma SSVFS..YLSDLEQITLAESQINKIEKSAFLGMT.NLLKLNLSK...............NFLGSIDSSTFQNLEKLEVL
grc-5ma YSVFS..CLSDLEQITLAESRINKIEKSAFLGMA.NLLKLNLSK...............NFLGNIDSNTFQNLEKLEVL
sma-5ma YSVFS..YLPDLEQITLAQSQINKIENNAFLGMT.NLLQLNLSK...............NVLGIINSETFQNLEKLEVL
sanh-5ma YSVFS..YLPDLEQITMAQSQINKIENNAFLGMT.NLLQLNLSK...............NVLGIINSETFQNLEKLEVL
san-5ma YSVFS..YLPDLEQITLAQSQINKIENNAFLGMT.NLLQLNLSK...............NVLGIINSETFQNLEKLEVL
srh-5ma YSVFS..YLPDLEQITLAQSQINKIENNAFWGMT.NLLQLNLSK...............NVLGIINSETFQNLEKLEVL
sgr-5ma YSVFS..YLPDLEQITLAQSQINKIENNAFLGMT.NLLQLNLSK...............NILGIINSDTFQNLEKLEVL
LTR5mb_ze KSVFS..HFTDLEQLTLAQNEINKIDDNAFWGLT.HLLKLNLSQ...............NFLGSIDSRMFENLDKLEVL
coc-5mb NSVFS..HFSDLEQITLAQNEINIIENNAFLGMT.NLQKLNLSN...............NFLGSVDSKTFQNLEKLEVL
gof-5mb DSVFS..HFPDLEQITLAQNEINIIENNAFWGLN.NLQKLNLSN...............NFLGSIDSKTFHNLEKLEVL
bsb-5b SSVFS..HFRDLEQITLAENEINIIEIDSFWGMT.NLLKLNLSK...............NFLGSIDSNTFQNLEKLEVL
grc-5mb NSVFS..HFQDLEQITLAENQINIIEIDAFWGMT.NLLKLNLSK...............NFLGNIDSNTFQNLKKLEVL
sma-5mb1 NSVFS..HFSDLEQITLAQNEINMIENNAFLGMT.DLQKLNLSK...............NFLGSIDSKTFQNLEKLEVL
san-5mb1 NSVFS..HFSDLEQITLAQNEINMIKNNAFLGMT.DLQKLNLSK...............NFLGSIDSKTFQNLEKLEVL
srh-5mb1 NSVFS..HFSDLEQITLAQNEINMIENNAFLGMT.DLQNLNLSN...............NFLGSIDSKTFQNLEKLEVL
sgr-5mb1 NSVFS..HFSDLEQITLAQNEINMIENNAFLGMT.DLQKLNLSK...............NFLGSIDSKTFQNLEKLEVL
sma-5mb2 NSVFSLFFFLDLEQITLAQNEINIIENNAFLGMT.NLQKLNLSN.....NFLVCLLYFKTHKPPLIDSKTFQNLEKLEVL
sanh-5mb2 NSVFS..HFSDLEQITLAQNST.LLKTMHFWCMT.NLQKLNLSN....NFLGMCLLYFKTHAPPLIDSKTFQNLEKLEVL
san-5mb2 .......VLQSLAPTFLNTPAFKFLVSKSLISWF.RDPRP..........IQLVCLLYFKTHKPPLIDSKTFQNLEKLEVL
srh-5mb2 NSVFSFFFFLDLEQITLAQNEINIIENDAFLGMT.NLQKLNLSNNFLGSIDSMCLLYFKTQAPPLIDSKTFQNLEKLEVL
sgr-5mb2 NSVFSLFFFLDLEQITLAQNEINIIENNAFLGMT.NLQKLNLSN.....NFLVCLLYFKTHKPPLIDSKTFQNLEKLEVL
rat-5ms YAVFS..PLREVEDITLAQNKINQIDRGAFWGLE.NLQRLNLSH...............NLIGEIYSYTFDNLPNILEL
chs-5ms YAVFS..PLREVEDITLAQNKINQIDRGAFWGLE.NLQRLNLSH...............NLIGEIYSYTFDNLPNIFEL
pis-5s NAVFS..PLREVEDITLAKNKINQIDRGAFWGLE.NLQRLNLSH...............NLIGEIYSYTFDNLPNILEL
csa-5s YAVFS..PLREVEDITLAQNKINQIDRGAFWGLE.NLQRLNLSH...............NLIGEIYSYTFDNLPNILEL
sas-5s YAVFS..SLREVEDITLAQNKINQIDRGAFWGLE.NLQRLNLSH...............NLIGEIYSYTFDNLPNILEL
sat-5s YAVFS..SLREVEDITLAQNKINQIDRGAFWGLE.NLQRLNLSH...............NLIGEIYSYTFDNLPNILEL
rat-5ma YAVFS..PLREVEDITIAQNKINQIDREAFFGLQ.NVQKLNLSH...............NLIGEIYSYTFDNLPNILEL
chs-5ma YAVFS..LLTEVEDMTIAQNKINQIDREAFFGLQ.NVQKLNLSH...............NLIGEIYSYTFDNLPNILEL
pis-5ma YAVFS..PLREVEGITIAQNKINQIDRDAFFGLQ.NVQKLNLSH...............NLIGEIYSYTFDNLPNILEL
csa-5ma ...............TIAQNKMNKI...EFFGL...LQWLNLSH...............NLIVEIYS.TFENLPSILEL
sas-5ma YAVFS..PLREVEDITIAQNKINQIDREAFFGLQ.NLQKLNLSH...............NLIGEIYSYTFDKLPNILEL
sat-5ma YAVFS..PLREVEDITIAQNKINQIDREAFFGLQ.NLQKLNLSH...............NLIGEIYSYTFDNLPNILEL
rat-5mb YAVFS..PLREVQDLTIAQNKMNKI...EFFGL....QWLNLSH...............NLIGEIYSYTFENLPSILEL
chs-5mb YAVFS..PLREVQDITIAQINMNKI...FFGLL....QWLNLSH...............NLIGEIYSYTFENLPSILEL
pis-5mb ...............TIAQNKMNKI...EFFGL...LQWLNLSH...............NLIGEIYS.TFENLPSILEL
csa-5mb YAVFS..PLREVEGITIAQNKINQIDREAFFGLQ.NVQKLNLSH...............NLIGEIYSYTFDNLPNILEL
sas-5mb YAVFN..PLREVQDITIAQNKINKIDRGEFFGLE.NLQWLNLSH...............NLIGEIYSYTFENLPNILEL
sat-5mb YAVFN..PLREVQDITIAQNKMNKIDRGEFFGLE.NLQWLNLSH...............NLIGEIYSYTFENLPNILKL

```
5m-human                                   β20                               β21                          TT  β22
              370       380       390       400       410       420       430
5m-human   DLQKNHIAIIQDQTFKFLEKLQTLDLRDNALTTIH.....FIPSIPDIFLSGNKLVTLP......KINLTANLIHLSENR
5m-pig     DLQKNHIGIIQDQTFRFLKKLNTLDLRDNALKTIQ.....FIPSIPTLFLGGNKLVTLP......NIRLTANFIHLSENR
inm-5m     DLSYNHIGVLGHQAFSDLPKLQELDLTGNSLRNLG...FPALLPNLNFLFLEDNRLTTLS..SILDLGNTSSYIDITGNR
zze-5m     DVSYNHIGILGYKAFRGLPNLRALYLTGNSLRNLG...FPESLPNLQWLLLADNKLQHLS..GISDLGVSSVHVDVRENR
lac-5s     DLSYNHIGILGYDSFNGLPNLKALYLTGNSLKDLG...FPASLPSLGYLMLNDNKLTSS..VGLSSFAHNVIYLNIQDNR
inm-5s     DLSHNHIGAVGYHSFSGLPNLKVLDLKGNSLRELG...SPASLPRLDYLILDDNKLTSV..YGLARFASNVSHLSVQNNR
zze-5s     DLSHNHIGVLGYRSFSGLPNLKSLYLTGNSLRDLG...FPESLPSLDHLLLNDNKLMPSSVISITGFASNVKYLNIQDNR
LTR5ma_ze  DLSYNHIWRLGSQSFQGLPNLLSLNLTGNSLGHVY...TFATLPKLEKLYLGDNNIQYVY..EIPNIAKHLKTLDLQFNQ
coc-5ma    DLSYNHIWMLGHQSFRGLPNLLNLNLTGNSLKYAH...TFATLPSLEKLYLGDNKITHAS..HLLNLAANLKTLYLQFNK
gof-5ma    DLSYNHIWRLGYQSFQGLPNLLILNLTGNSIKSAH...TFATLPNLEKLYLGVNKFTHAF..SLLNIGTNLKTLYLQFNK
bsb-5ma    DLSYNHIWMLGYKSFQGLPNLLNLNLRGNALKHVH...LFAAIPNLEKLYLGDNKISSVY..YLNKISKYLTTLDLESNQ
grc-5ma    DLSYNHIWMLGYESFRGLPNLLNLNLTGNALKHLH...AFATLPRLEKLYLGDNKILSVF..YLIKISKYLTTLYLEHNI
sma-5ma    DLSYNHIWKLGHQSFQGLPNLLNLNLTGNSLKYAH...TFASLPSLEKLYLGDNKMTHAS..NLLNIATNLKTLYLQFNK
sanh-5ma   DLSYNHIWKLGHQSFQGLPNLLDLNLTGNSLKYAH...TFASLPSLEKLYLGDNKITHAS..NLLNIATNLKTLYLQFNK
san-5ma    DLSYNHIWKLGHQSFQGLPNLLNLNLTGNSLKYAH...TFASLPSLEKLYLGDNKITHAS..YLLNIATNLKTLYLQFNK
srh-5ma    DLSYNHIWTLGHQSFQGLPNLLNLNLTGNSLKYAH...TFASLPSLEKLYLGDNKITHAS..NLLNIATNLKTLYLQFNK
sgr-5ma    DLSYNHIWKLGHQSFQGLTNLLNLNLTGNSLKYAH...TFASLPSLEKLYLGDNKITHVS..NLLNIATNLKTLYLQFNK
LTR5mb_ze  DLSYNHIRALGDQSFLGLPNLRKLNLTGNAVESVH...TFAALPNLNKLYLGKNRISSVS..SLPNIAHNLSTLDLEFNK
coc-5mb    DLSYNHIRVLGDQSFRGLPSLLELNLTGNSLESVH...EFATLPNLKKLYLGENRILSLS..SLPNIAKNITTLDLEFNR
gof-5mb    DLSYNHIRVLGDQSFQGLTSLINLNLTENALESVN...EFATLPNLKKLYLGDNSISSLS..SLPNIAKNLTTLDLEFNR
bsb-5b     DLSYNHIRVLGDKSFQGLPSLLNLNLTGNALESVH...EFATLPNLKIIYLGDNKISSLS..SLPNIAKNLTTLDLEFNK
grc-5mb    DLSYNHIRVLGDKSFQGLPSLLNLNLTGNALESVH...EFATLPNLKIIYLGENRISSLS..SLPNIAKNLTTLDLEMNK
sma-5mb1   DLSYNHIRVLGDQSFQGLPSLLNLNLTENALESIH...EFATLPNLKKLYLGENRISSLS..SLPNIAKNLTTLDLEFNR
san-5mb1   DLSYNHIRVLGDQSFQGLPSLLNLNLTENALESIH...EFATLPNLKKLYLGENRISSLS..SLPNIAKNLTTLDLEFNR
srh-5mb1   DLSYNHIRVLGDQSFQGLPSLLNLNLTENALESIH...KFATLPNLKKLYLGENRISSLS..SLPNIAKNLTTLDLEFNR
sgr-5mb1   DLSYNHIRVLGEQSFQGLPCLLNLNLTENALESIH...EFATLPNLKKLYLGENRISSLS..SLPNIAKNLTTLDLEFNR
sma-5mb2   DLSYNYVRVLGDQSFLGLPNLLNLNLR.AALESVH...EFATLANLKKLYLGENRISSLS..ILPNISKNLTTLDLEFNR
sanh-5mb2  DLSYNYIRVLGDQSFQGLPNLLNLNLTGNALESVH...EFATLANLKKLYLGENRISSLS..ILPNISKNLTTLDLEFNR
san-5mb2   DLSYNYVRVLGDQSFLGLPNLLNLNLR.AALESVH...EFATLANLKKLYLGENRISSLS..ILPNISKNLTTLDLEFN.
srh-5mb2   DLSYNYIRIIY.QSFQGLPNLLNLNLTGNALESVH...EFATLANLKKLYLGENRISSLS..ILPNIS.NLTTLDLEFNR
sgr-5mb2   DLSYNYVRVLGDQSFLGLPNLLNLNLR.AALESVH...EFATLANLKKLYLGENRISSLS..ILPNISKNLTTLDLEFN.
rat-5ms    DLSYNHIGALGYQAFTGLPNLQILDLTGNSIRQLGTYGYLAPLPNLQLLNLADNKITSLE..GLLGFANSTIILNVQNNR
chs-5ms    DLSYNHIGALGYQAFTGLPHLQILDLTGNSIRQLGTYGYLAPLPNLQLLHLADNKITSLE..GLLGFANSTIILNVQNNR
pis-5s     DLSYNHIGALGYQAFTGLPNLQILDLTGNSIRQLGTYGYLAPLPNLQLLHLADNKITSLE..GLLGFANSTIILNVQNNR
csa-5s     DLSYNHIGALGYQAFTGLPNLQILDLTGNSIRQLGTYGYLAPLPNLQLLHLADNKITSLE..GLLGFANSTIILNVQNNR
sas-5s     DLSYNHIGALGYQAFTGLPNLQILDLTGNSIRQLGTYGYLAPLPNLQLLHLADNKITSLE..GLLGFANSTIILNVQNNR
sat-5s     DLSYNHIGALGYQAFTGLPNLQILDLTGNSIRQLGTYGYLAPLPNLQLLHLADNKITSLE..GLLGFANSTIILNVQNNR
rat-5ma    DISYNHIGALGYQAFKGLPNLQVLDLTGNSIRGLGTYGSLAPLPNIQLLRLADNKITSLE..GLSVFAPNTIILNVQNNR
chs-5ma    DLSYNHIGALGYQAFKGLPNLQVLDLTGNSIRELGTYGSLAPLPNIQLLRLADNKITSLE..GLSVFAPNTIILNVQNNR
pis-5ma    DVSYNHIGALGYQAFKGLPNLQVLDLTGNSIRELGTYGSLAPLPNIQLLRLADNKITSLD..GLSVFAPNTIILNVQNNR
csa-5ma    DLSYNHIGALGYQAFTGLPHLQVLDITEHFIRDLGTNGYLAPLPNIQLLCLTDNKITSLE..GLAGFADSTIILNIQNNR
sas-5ma    DLSYNHIGALGYHAFKGLPNLQFLDLTGNSIRELGTYGSLAPLPNIQHLRLADNKITSLE..GLSVFANSTIILNVQNNR
sat-5ma    DLSYNHIGALGYHAFKGLPNIQFLDLTGNSIRELGTYGSLAPLPNIQHLRLADNKITSLA..GLSVFANSTIILNVQNNR
rat-5mb    DLSYNHIGALGYQAFTGLPHLQVLDLTENFFRDLGTNGYLAPLPNIQLLRLTDSKITSLE..GLAGFADSTIILNIQNNR
chs-5mb    DLSYNHIGALGYQAFTGLPHLQVLDLTEN.FRDLGTNGYLAPFPNIQLLRLTDNKITSLE..GLAGFANSTIILNIQNNR
pis-5mb    DLSYNHIGALGYQAFTGLPHLQVLDITENFICDLGTNGYLAPLPNIQLLCLTDNKITSLE..GLSGFADSTIMLNIQNNR
csa-5mb    DLSYNHIGALGYQAFKGLPNLQVLDLTGNSIRELGTYGSLAPLPNIQLLRLADNKITSLD..GLSVFAPNTIILNVQNNR
sas-5mb    DLSYNHIGALGYQAFTGLPHLQVLDLTENSIRDLGTNGYLAPLPNIQLLHLTDNKITSLE..GLAGFADSTIILNIQNNR
sat-5mb    DLSYNHIGALGYQAFTGLPHLQVLDLTENSIRDLGTNGYLPQLPNIQLLHLTDNKITSLE..GLAGFADSTIILNIQNNR
```

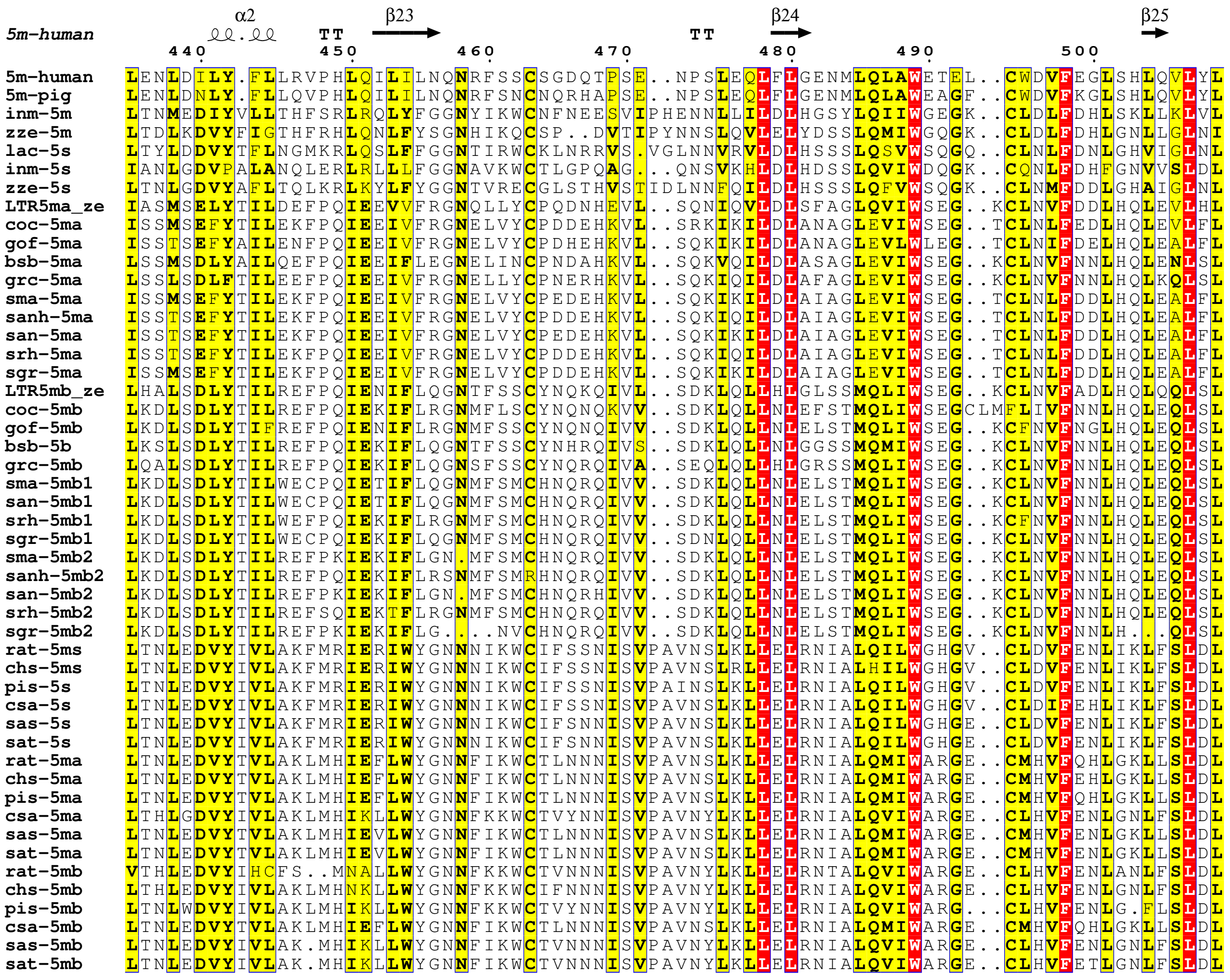

5m-human
α2
β23
β24
β25
TT
TT
440
450
460
470
480
490
500
5m-human
5m-pig
inm-5m
zze-5m
lac-5s
inm-5s
zze-5s
LTR5ma_ze
coc-5ma
gof-5ma
bsb-5ma
grc-5ma
sma-5ma
sanh-5ma
san-5ma
srh-5ma
sgr-5ma
LTR5mb_ze
coc-5mb
gof-5mb
bsb-5b
grc-5mb
sma-5mb1
san-5mb1
srh-5mb1
sgr-5mb1
sma-5mb2
sanh-5mb2
san-5mb2
srh-5mb2
sgr-5mb2
rat-5ms
chs-5ms
pis-5s
csa-5s
sas-5s
sat-5s
rat-5ma
chs-5ma
pis-5ma
csa-5ma
sas-5ma
sat-5ma
rat-5mb
chs-5mb
pis-5mb
csa-5mb
sas-5mb
sat-5mb

```
                α3                   β26                   β27                     β28
5m-human       ℓℓℓℓ        TT
            510        520        530        540        550        560        570        580
5m-human    NHNYLNSLPPGVFSHLTALRGLSLNSNRLTVLSHNDLPANLEILDISRNQLLAPNPDVFVSLSVLDITH...NKFICECE
5m-pig      NNNYLNFLPPGVFRHLTALRGLSLSYNRLTVLYPGDLPAHLEVLDLSRNQLLSPDPDLFTSLSAVDLSH...NKFVCDCE
inm-5m      SFNSLKVLPPGIFLGLTSIVDIDLSFNSLTYLETDVFPTSLEVLRLSNNFLATPDPKVFQSLTFLGLSG...NPFYCDCS
zze-5m      SFNSLTTLPPGIFRGLSSIIELDLSSNALTYLQPDVFPFSLKTLYLSNNFLAFPDPTNFLSLSFLSLAE...NRFHCSCN
lac-5s      SSNALQSLPQGIFKGLTAIVQMDLSSNALTYLQPDALPKSLKILNLSNNFIASPDPATFRSLSYLNLNT...NRFHCNHN
inm-5s      SSNRLRALPRKAFQGLTSVEKVNLSSNALTYLHPGELPGTLKSLDLSGNFLASPDPAVFRSLSVVGLSM...NRFLCSPE
zze-5s      SFNALQFLPQGIFKGLTSVKEMDLSSNSLTYLQPDIFPRSLKVLNLSNNFIASPDPATFRSLDILDLNM...NRFHCDSS
LTR5ma_ze   SSNLLQSLPKDIFKDLTSLLILDLSFNSLKYLPTDVFPKTLQVLHLDYNSIYSVDPNLFSTLSYLSLMN...NDFRCDCD
coc-5ma     SSNRLQSLPKDIFKDLTSLIFLDLSSNSLKYLPNGIFPKSLQYLNLEFNSVYSVDPNLFSTLSFLSLLG...NDFNCDCN
gof-5ma     NSNRLQSLPKDVFKDLTSLIFLDLSSNSLKYLPNGIFPKSLQVLNLEFNSVYAVDPNLFSTLSYLSLLG...NDFNCDCN
bsb-5ma     SHNLLRSLPKDIFKDLNSLYHLDLSFNSLKYLPNGIFPESLQVLYLAYNSIYSVDPNLFSTLSYLSLEK...NDFRCDCN
grc-5ma     SHNLLQSLPKDIFKDLTSLYFLDLSFNSLKYLPNGIFPESLQILNLEYNSIYSVDPNLFSTLSYLSLIK...NDFRCDCK
sma-5ma     SSNRLQSLPKDIFKDLTSLIFLDLSSNSLKYLPNGIFPKSLQYLNLEYNSVYSVDPNLFSTLSYLSLLG...NGFNCDCN
sanh-5ma    SSNRLQSLPKDIFKDLNSLIFLDLSSNSLKYLPNGIFPTNLQYLNLEYNSVYSVDPNLFSTLSYLSLLG...NGFNCDCN
san-5ma     SSNRLQSLPKDIFKDLTSLIFLDLSSNSLKYLPNGIFPKSLQYLNLEYNSVYSVDPNLFSTLSYLSLLG...NGFNCDCN
srh-5ma     SSNRLQSLPKDIFKDLTSLIFLDLSSNSLKYLPNGIFPKSLQYLNLEYNSVYSVDPNLFSTLSYLSLLG...NGFNCDCN
sgr-5ma     SSNRLQSLPKDIFKDLTSLIFLDLSSNSLKYLPNGIFPKSLQYLNLEYNSVYSVDPNLFSTLSYLSLLG...NGFNCDCN
LTR5mb_ze   TANGLQSLPKDIFKDLTSLFFLDLSFNSLKYLPTDVFPKSLQILNLDYNSIYSVDPNLFSTLSYLSLMN...NDFRCDCD
coc-5mb     ASNGLQSLPKEIFKDLTSLFFLDLSFNSLKYLPNGIFPKSLQILNLEYNSIYSVDPNLFSTLSYLSLLK...NQFRCDCN
gof-5mb     ASNGLQSLPKDIFKDLTSLFFLDLSFNSLKYLPNGIFPKSLQILNLEYNSIYSVDPNLFSTLSYLSLLK...NQFRCDCN
bsb-5b      TANGLQSLPKDIFKDLTSLWILDLSFNSLKYLPNGIFPESLQILNLEYNSIYSVDPNLFSTLSYLSLLSLKKNDFRCDCN
grc-5mb     TANGLQSLPKDIFKDLTSLFFLDLSFNSLKYLPNGIFPESLQILNLEYNSIYSVDPNLFSTLSYLSLI...KNDFRCDCN
sma-5mb1    ASNGLQSLPKDIFKDLTSLFFLDLSFNSLKYLPNGIFPKSLQILNLEYNSIYSVDPNLFSALSYLSLIK...NQFRCDCN
san-5mb1    ASNGLQSIPKDIFKDLTSLFFLDLSFNSLKYLPNGIFPKSLQILNLEYNSIYSVDPNLFSALSYLSLIK...NQFRCDCN
srh-5mb1    ASNGLQSLPKDIFKDLTSLFFLDLSFNSLKYLPNGIFPKSLQILNLEYNSIYSVDPNLFSTLSYLSLIK...NQFRCDCN
sgr-5mb1    ASNGLQSLPKDIFKDLTSLFFLDLSFNSLKYLPNGIFPKSLQILNLEYNSIYSVDPTLFSALSYLSLIK...NQFRCDCN
sma-5mb2    ASNGLQSIPKDIFKDLTSLIFLDLSFNSLKHLLNGIFPKSLLILKLEYNSIYSVGPNLFSTLSYLSLMK...KQFRCDCS
sanh-5mb2   ASNGLQSLPKDIFKDLTSLIFLDLSFNSLKHLPNGIFPKSLQILKLEYNSIYSVDPNLFIALS...LMK...NQFRCDCS
san-5mb2    ASNGLQSIPKDIFKDLTSLIFLDLSFNSLKHLLNGIFPKSLLILKLEYNSIYSVGPNLFSTLSYLSLMK...KQFRCDCS
srh-5mb2    ASNGLQSLPKDIFKDLTSLIFLDLSFNSLKHLPNGIFPESLQILKSG.........LFSTLSYLSLMK...NQFHCDCS
sgr-5mb2    ASNGLQSIPKDIFKDLTSLIFLDLSFNSLKHLPNGIFPKSLLILKLEYNSIYSVGPNLFSTLS...LMK...NQFRCDCS
rat-5ms     SFNSLRALPDGIFKGLVSLEEMDLSFNSLTYLQPDIFPASLKTVDLSYNFLSSPDPAAFSSLSWINLYR...NRFHCDGG
chs-5ms     SFNSLRALPDGIFKGLVSLEEMDLSFNSLTYLQPDIFPASLKTVDLSYNFLSSPDPATFSSLSWINLYR...NRFHCDGG
pis-5s      SFNSLRALPDGIFKGLVSLEEMDLSFNSLTYLQPDIFPASLKTVDLSYNFLSSPDPATFSSLSWINLYR...NRFHCDRG
csa-5s      SFNSLRALPDGIFKGLVSLEEMDLSFNSLTYLQPDIFPASLKTVDLSYNFLSSPDPATFSSLSWINLYR...NRFHCDGG
sas-5s      SFNSLRALPNGIFKGLVSLEEMDLSFNSLTYLQPDIFPVSLKTVDLSYNFLSSPDPAAFRSLSWINLYR...NRFHCDGG
sat-5s      SFNSLRALPNGIFKGLVSLEEMDLSFNSLTYLQPDIFPASLKTVDLSYNFLSSPDPAAFRSLSWINLYR...NRFHCDGG
rat-5ma     SFNSLQALPDGIFKGLISLEEMDLHFNSLTYLKQDIFPESLKTVDLSYNFLASPDPEAFHSLSWINLYR...NQFHCDCG
chs-5ma     SFNSLQALPDGIFKGLISLEEMDLHFNSLTYLKPDIFPESLKTVDLSYNFLASPDPEAFHSLSWINLYR...NQFHCDCG
pis-5ma     SFNSLQALPDGIFKGLISLEEMDLHFNSLTYLKPDIFPESLKTVDLSYNFLASPDPEAFHSLSWINLYR...NQFHCDCG
csa-5ma     SLNSLQALPDGIFKGLISLEE.DLHFNFLTYLQPDIFPETLKTLHLSY.FLASPDPAAFHSLSWINLYR...NRFHCDCG
sas-5ma     SFNSLQTLPDGIFKGLISLEEMDLHFNSLTYLKPDIFPESLKTVDLSYNFLASPDPVAFHSLSWINLYR...NQFHCDCG
sat-5ma     SFNSLQTLPDGIFKGLISLEEMDLHFNSLTYLKPDIFPESLKTVDLSYNFLASPDPVAFHSLSWINLYR...NQFHCDCG
rat-5mb     .LNSLQALPDGIFKGLISLEE.DLHFNFLTYLQSDIFPETLKTLHLSY.FLASPDPAAFHSLSWINLCR...NRFHCDCG
chs-5mb     SLNSLQALPDGIFKGLISLEEMDLHFNSLTYLKPDIFPESLKTVDLSYNFLASPDPEAFHSLSWINLYR...NRFHDST.
pis-5mb     SLNSLQALPDGIFKGLISLEE.MISTSTSSHLQPDIFPETLKTLHLSY.FLASPDPAASHSLSWINLYR...NRFHCDCG
csa-5mb     SFNSLQALPDGIFKGLISLEEMDLHFNSLTYLKPDIFPESLKTVDLSYNFLASPDPEAFHSLSWINLYR...NQFHCDCG
sas-5mb     SLNSLQALPDGIFKGLISLEE.DLHFNFLTYLQPDIFPETLKTLHLSY.FLASPDPAAFHSLSWINLYR...NRFHCDCG
sat-5mb     SLNSLQALPDGIFKGLISLEE.DLHFNFLTYLQPDIFPETLKTLHLSY.FLASSDPAAFHSLSWINLYR...NRFH....
                                                                                     1 2
```

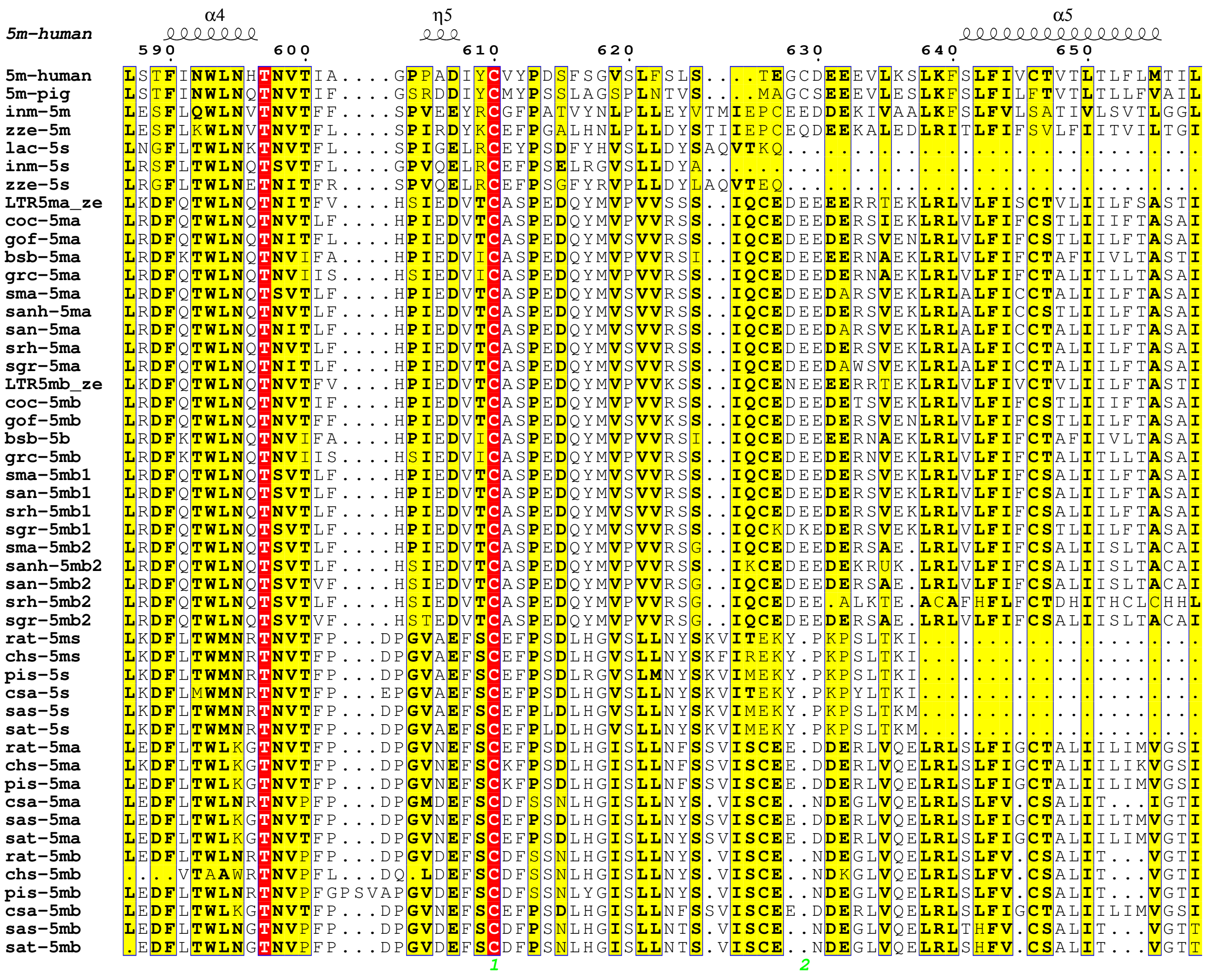
5m-human
α4
η5
α5
590
600
610
620
630
640
650
5m-human
5m-pig
inm-5m
zze-5m
lac-5s
inm-5s
zze-5s
LTR5ma_ze
coc-5ma
gof-5ma
bsb-5ma
grc-5ma
sma-5ma
sanh-5ma
san-5ma
srh-5ma
sgr-5ma
LTR5mb_ze
coc-5mb
gof-5mb
bsb-5b
grc-5mb
sma-5mb1
san-5mb1
srh-5mb1
sgr-5mb1
sma-5mb2
sanh-5mb2
san-5mb2
srh-5mb2
sgr-5mb2
rat-5ms
chs-5ms
pis-5s
csa-5s
sas-5s
sat-5s
rat-5ma
chs-5ma
pis-5ma
csa-5ma
sas-5ma
sat-5ma
rat-5mb
chs-5mb
pis-5mb
csa-5mb
sas-5mb
sat-5mb
1
2

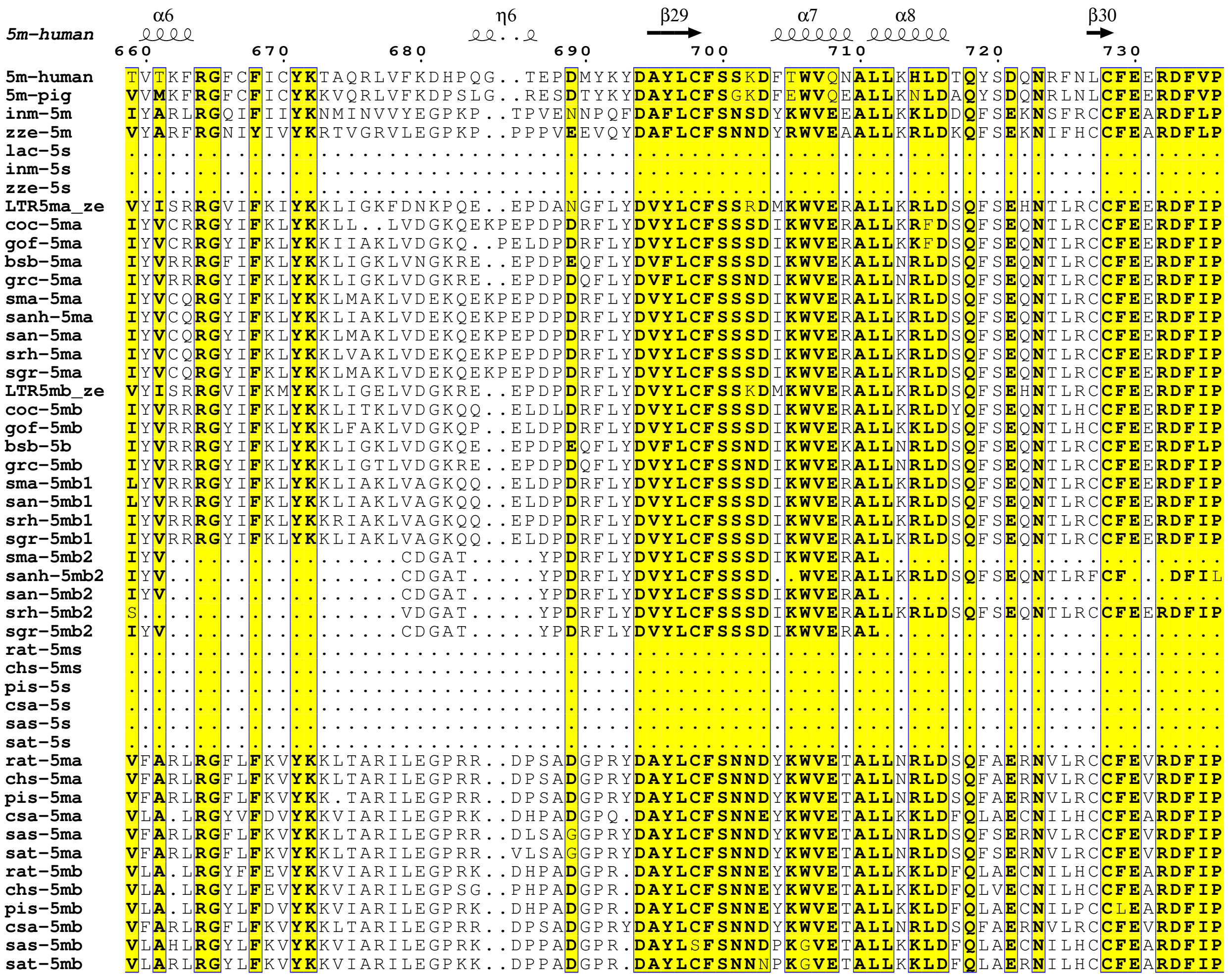
5m-human
α6
η6
β29
α7
α8
β30

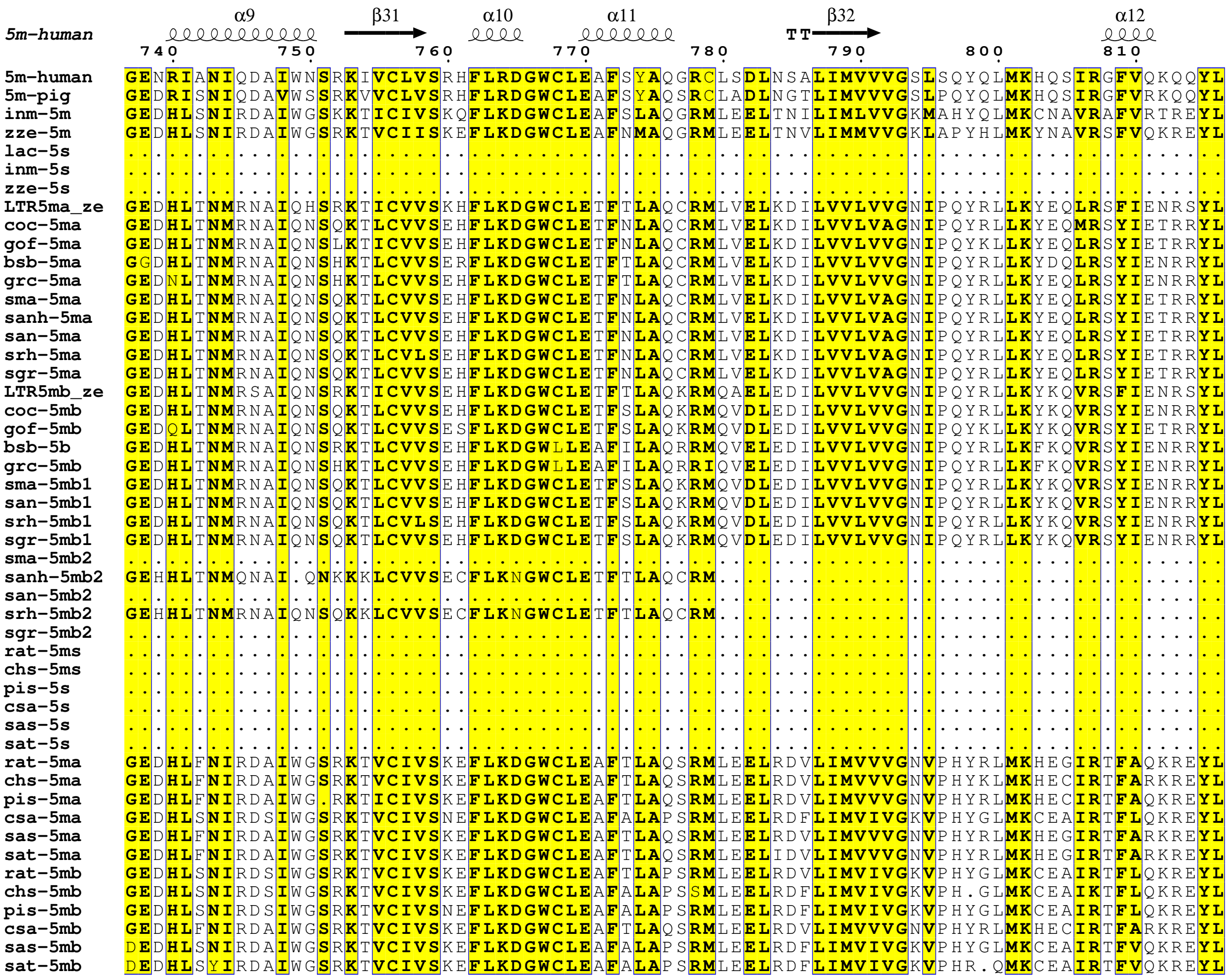

α9 β31 α10 α11 TT β32 α12
5m-human
740 750 760 770 780 790 800 810
5m-human GENRIANIQDAIWNSRKIVCLVSRHFLRDGWCLEAFSYAQGRCLSDLNSALIMVVVGSLSQYQLMKHQSIRGFVQKQQYL
5m-pig GEDRISNIQDAVWSSRKVVCLVSRHFLRDGWCLEAFSYAQSRCLADLNGTLIMVVVGSLPQYQLMKHQSIRGFVRKQQYL
inm-5m GEDHLSNIRDAIWGSKKTICIVSKQFLKDGWCLEAFSLAQGRMLEELTNILIMLVVGKMAHYQLMKCNAVRAFVRTREYL
zze-5m GEDHLSNIRDAIWGSRKTVCIISKEFLKDGWCLEAFNMAQGRMLEELTNVLIMMVVGKLAPYHLMKYNAVRSFVQKREYL
lac-5s ....
inm-5s ....
zze-5s ....
LTR5ma_ze GEDHLTNMRNAIQHSRKTICVVSKHFLKDGWCLETFTLAQCRMLVELKDILVVLVVGNIPQYRLLKYEQLRSFIENRSYL
coc-5ma GEDHLTNMRNAIQNSQKTLCVVSEHFLKDGWCLETFNLAQCRMLVELKDILVVLVAGNIPQYRLLKYEQMRSYIETRRYL
gof-5ma GEDHLTNMRNAIQNSLKTICVVSEHFLKDGWCLETFNLAQCRMLVELKDILVVLVVGNIPQYKLLKYEQLRSYIETRRYL
bsb-5ma GGDHLTNMRNAIQNSHKTLCVVSERFLKDGWCLETFTLAQCRMLVELKDILVVLVVGNIPQYRLLKYDQLRSYIENRRYL
grc-5ma GEDNLTNMRNAIQNSHKTLCVVSEHFLKDGWCLETFTLAQCRMLVELKDILVVLVVGNIPQYRLLKYEQLRSYIENRRYL
sma-5ma GEDHLTNMRNAIQNSQKTLCVVSEHFLKDGWCLETFNLAQCRMLVELKDILVVLVAGNIPQYRLLKYEQLRSYIETRRYL
sanh-5ma GEDHLTNMRNAIQNSQKTLCVVSEHFLKDGWCLETFNLAQCRMLVELKDILVVLVAGNIPQYRLLKYEQLRSYIETRRYL
san-5ma GEDHLTNMRNAIQNSQKTLCVVSEHFLKDGWCLETFNLAQCRMLVELKDILVVLVAGNIPQYRLLKYEQLRSYIETRRYL
srh-5ma GEDHLTNMRNAIQNSQKTLCVLSEHFLKDGWCLETFNLAQCRMLVELKDILVVLVAGNIPQYRLLKYEQLRSYIETRRYL
sgr-5ma GEDHLTNMRNAIQNSQKTLCVVSEHFLKDGWCLETFNLAQCRMLVELKDILVVLVAGNIPQYRLLKYEQLRSYIETRRYL
LTR5mb_ze GEDHLTNMRSAIQNSRKTICVVSEHFLKDGWCLETFTLAQKRMQAELEDILVVLVVGNIPQYRLLKYKQVRSFIENRSYL
coc-5mb GEDHLTNMRNAIQNSQKTLCVVSEHFLKDGWCLETFSLAQKRMQVDLEDILVVLVVGNIPQYRLLKYKQVRSYIENRRYL
gof-5mb GEDQLTNMRNAIQNSQKTLCVVSESFLKDGWCLETFSLAQKRMQVDLEDILVVLVVGNIPQYKLLKYKQVRSYIETRRYL
bsb-5b GEDHLTNMRNAIQNSRKTLCVVSEHFLKDGWLLEAFILAQRRMQVELEDILVVLVVGNIPQYRLLKFKQVRSYIENRRYL
grc-5mb GEDHLTNMRNAIQNSHKTLCVVSEHFLKDGWLLEAFILAQRRIQVELEDILVVLVVGNIPQYRLLKFKQVRSYIENRRYL
sma-5mb1 GEDHLTNMRNAIQNSQKTLCVVSEHFLKDGWCLETFSLAQKRMQVDLEDILVVLVVGNIPQYRLLKYKQVRSYIENRRYL
san-5mb1 GEDHLTNMRNAIQNSQKTLCVVSEHFLKDGWCLETFSLAQKRMQVDLEDILVVLVVGNIPQYRLLKYKQVRSYIENRRYL
srh-5mb1 GEDHLTNMRNAIQNSQKTLCVLSEHFLKDGWCLETFSLAQKRMQVDLEDILVVLVVGNIPQYRLLKYKQVRSYIENRRYL
sgr-5mb1 GEDHLTNMRNAIQNSQKTLCVVSEHFLKDGWCLETFSLAQKRMQVDLEDILVVLVVGNIPQYRLLKYKQVRSYIENRRYL
sma-5mb2 ....
sanh-5mb2 GEHHLTNMQNAI.QNKKKLCVVSECFLKNGWCLETFTLAQCRM.....................................
san-5mb2 ....
srh-5mb2 GEHHLTNMRNAIQNSQKKLCVVSECFLKNGWCLETFTLAQCRM.....................................
sgr-5mb2 ....
rat-5ms ....
chs-5ms ....
pis-5s ....
csa-5s ....
sas-5s ....
sat-5s ....
rat-5ma GEDHLFNIRDAIWGSRKTVCIVSKEFLKDGWCLEAFTLAQSRMLEELRDVLIMVVVGNVPHYRLMKHEGIRTFAQKREYL
chs-5ma GEDHLFNIRDAIWGSRKTVCIVSKEFLKDGWCLEAFTLAQSRMLEELRDVLIMVVVGNVPHYKLMKHECIRTFARKREYL
pis-5ma GEDHLFNIRDAIWG.RKTICIVSKEFLKDGWCLEAFTLAQSRMLEELRDVLIMVVVGNVPHYRLMKHECIRTFAQKREYL
csa-5ma GEDHLSNIRDSIWGSRKTVCIVSNEFLKDGWCLEAFALAPSRMLEELRDFLIMVIVGKVPHYGLMKCEAIRTFLQKREYL
sas-5ma GEDHLFNIRDAIWGSRKTVCIVSKEFLKDGWCLEAFTLAQSRMLEELRDVLIMVVVGNVPHYRLMKHEGIRTFARKREYL
sat-5ma GEDHLFNIRDAIWGSRKTVCIVSKEFLKDGWCLEAFTLAQSRMLEELIDVLIMVVVGNVPHYRLMKHEGIRTFARKREYL
rat-5mb GEDHLSNIRDSIWGSRKTVCIVSKEFLKDGWCLEAFTLAPSRMLEELRDVLIMVIVGKVPHYGLMKCEAIRTFLQKREYL
chs-5mb GEDHLSNIRDSIWGSRKTVCIVSKEFLKDGWCLEAFALAPSSMLEELRDFLIMVIVGKVPH.GLMKCEAIKTFLQKREYL
pis-5mb GEDHLSNIRDSIWGSRKTVCIVSNEFLKDGWCLEAFALAPSRMLEELRDFLIMVIVGKVPHYGLMKCEAIRTFLQKREYL
csa-5mb GEDHLFNIRDAIWGSRKTVCIVSKEFLKDGWCLEAFTLAQSRMLEELRDVLIMVVVGNVPHYRLMKHECIRTFARKREYL
sas-5mb DEDHLSNIRDAIWGSRKTVCIVSKEFLKDGWCLEAFALAPSRMLEELRDFLIMVIVGKVPHYGLMKCEAIRTFVQKREYL
sat-5mb DEDHLSYIRDAIWGSRKTVCIVSKEFLKDGWCLEAFALAPSRMLEELRDFLIMVIVGKVPHR.QMKCEAIRTFVQKREYL

```
                        α13
5m-human
              820       830       840       850
5m-human      RWPEDLQDVGWFLHKLSQQILKKEKEKKKDNNIPLQTVATIS...........
5m-pig        RWPEDLQDVSWFLNKLSQYILKKVKEKKKDSDIQLQSVTIS............
inm-5m        TWPEDPQDLDWFYEKLSLQILKDTK............................
zze-5m        VWPEDPQDLGWFYERLISQILKDTK............................
lac-5s        .....................................................
inm-5s        .....................................................
zze-5s        .....................................................
LTR5ma_ze     VWPDDGQDLEWFYDQLLHKIIRNTKVKQTNIKEKDKVDENNPEAADVQADTAV
coc-5ma       MWPDDSQDLEWFYDQLLDKIRKNTKIKQTNAKEKAKEVKNNPEAVNVYADTAV
gof-5ma       QWPDDSQDLEWFYDQLLHKIRNNTKVTQIDAKEKDKEVKNNPEAANVYADTAV
bsb-5ma       LWPDDSQDLDWFYDQLLHKIRKNTKVKQTNIKVNEG..EKNLDAANLQADTAV
grc-5ma       LWPDDSQDLEWFYDQLLHKIRKNTKVKQTNTKVNEG..EKNLDAANVQADTAV
sma-5ma       MWPDDNQDLEWFYDQLLHKIRKNTKVKQTNAKEKDKEVKNNPEAANVHADTAV
sanh-5ma      MWPDDNQDLEWFYDQLLHKIRKNTKVKQTNAKEKDK.VKNNPEAANVHADTAV
san-5ma       MWPDDNQDLEWFYDQLLHKIRKNTKIKQTNAKEKDK.VKINPEAANVHADTAV
srh-5ma       TWPDDNQDLEWFYDQLLHKIRKNTKVKQTNAKEKDKEVKNNPEAANVHADTAV
sgr-5ma       MWPDDNQDLEWFYDQLLHKIRKNTKVKQTNAKEKDKEVKNNPEAANVHADTAV
LTR5mb_ze     VWPDDCQDLEWFYDQLLHKIRKDIKINQT...TKEIK....REEANFNTNTAV
coc-5mb       LWPDDSQDLEWFYDQLLHKIKKDTKVKQTNQPTEQIK....TDATNVHANTEV
gof-5mb       MWPDDSQDLEWFYDQLLYKIKKDTKVKQANQPTEQTK....TDATNVHANTEV
bsb-5b        LWPDDSQDLDWFYDQLLHKIRKDTKVKQTNQPNKQTK....PEALNVHANTAV
grc-5mb       LWPDDSQDLDWFYDQLLHKIRKDTKVKQTNQPTKQTK....PEALNVHANTSV
sma-5mb1      LWPDDSQDLDWFYDQLLHKIKKDTKVKQTNQPTGQTK....ADATNIHANTAV
san-5mb1      LWPEDSQDLDWFYDQLLHKIKKDTKVKQTNQPTGQTK....ADANT.......
srh-5mb1      LWPDDSQDLDWFYDQLLHKIKKDTKVKQTNQPTGQTK....ADATNIHANTAV
sgr-5mb1      LWPDDSQDLDWFYDQLLHKIKKDTKVKQTNQPTGQTK....ADANT.......
sma-5mb2      .....................................................
sanh-5mb2     .....................................................
san-5mb2      .....................................................
srh-5mb2      .....................................................
sgr-5mb2      .....................................................
rat-5ms       .....................................................
chs-5ms       .....................................................
pis-5s        .....................................................
csa-5s        .....................................................
sas-5s        .....................................................
sat-5s        .....................................................
rat-5ma       QWPEDTQDIQWFYEKLMSKILKD...KKYTSKDNNRDITLVNMT.VGT.....
chs-5ma       QWPEDTQDIQWFYEKLMSKILKD...KKNTSKDNNGDITLVNMT.VGT.....
pis-5ma       QWPEDTQDIQWFYEKLMSKILKD...KKNTAKDNNGDITLVNMT.VGT.....
csa-5ma       QRPEDTQDIEWFYEKLMSKILNDRKIKKDTAKDNNGDITLVNMQAVGT.....
sas-5ma       QWPEDTQDIQWFYEKLMSKILKD...KKNTAKDNNGDITLVNMT.VGT.....
sat-5ma       QWPEDTQDIQWFYEKLMSKILKD...KKNTAKDNNGEITLVNMT.VGT.....
rat-5mb       QRPEDTQDIEWFYEKLMSKILNDRKIKKDTAKDNNGDITLVNMQAVGT.....
chs-5mb       QRPEDTHDI.WFYEKLMSKILNDRKIKKDTAKDKNGDITLVNMQAVGT.....
pis-5mb       QRPEDTQDIEWFYEKLMSKNLNDRKIKKDTAKDNNGDITLVNMQAVGT.....
csa-5mb       QWPEDTQDIQWFYEKLMSKILKD...KKNTSKDNNGDITLVNMT.VGT.....
sas-5mb       QRPEDTQDIEWFY........................................
sat-5mb       QRLEDTQDIEWFYEKLMSKILNNRKIKKDTAKDNNGDITLVNMQAVGT.....
```
